# Advanced Phytochemical-Based Nanocarrier Systems for the Treatment of Breast Cancer

**DOI:** 10.3390/cancers15041023

**Published:** 2023-02-06

**Authors:** Vivek P. Chavda, Lakshmi Vineela Nalla, Pankti Balar, Rajashri Bezbaruah, Vasso Apostolopoulos, Rajeev K. Singla, Avinash Khadela, Lalitkumar Vora, Vladimir N. Uversky

**Affiliations:** 1 Department of Pharmaceutics and Pharmaceutical Technology, L. M. College of Pharmacy, Ahmedabad 380009, Gujarat, India; 2 Department of Pharmacy, Koneru Lakshmaiah Education Foundation, Vaddeswaram, Guntur 522302, Andhra Pradesh, India; 3 Pharmacy Section, L. M. College of Pharmacy, Ahmedabad 380009, Gujarat, India; 4 Department of Pharmaceutical Sciences, Faculty of Science and Engineering, Dibrugarh University, Dibrugarh 786004, Assam, India; 5 Institute for Health and Sport, Victoria University, Melbourne, VIC 3030, Australia; 6 Institutes for Systems Genetics, Frontiers Science Center for Disease-Related Molecular Network, West China Hospital, Sichuan University, Xinchuan Road 2222, Chengdu 610064, China; 7 School of Pharmaceutical Sciences, Lovely Professional University, Phagwara 144411, Punjab, India; 8 Department of Pharmacology, L. M. College of Pharmacy, Ahmedabad 380009, Gujarat, India; 9 School of Pharmacy, Queen’s University Belfast, 97 Lisburn Road, Belfast BT9 7BL, UK; 10 Department of Molecular Medicine, Byrd Alzheimer’s Research Institute, Morsani College of Medicine, University of South Florida, Tampa, FL 33613, USA

**Keywords:** phytochemicals, nanocarriers, breast cancer, chemotherapy, drug resistance

## Abstract

**Simple Summary:**

Breast cancer is a concern for the healthcare system. Even with the advancement of science and technology, the current system for therapeutics and diagnostics seems to have numerous pitfalls. Phytochemical-mediated nanocarriers come into the picture to outrange the drawbacks of the conventional breast cancer management method. Phytochemicals have been a useful tool since time immemorial, and developing a sophisticated fusion of these chemicals with nanocarrier enhanced its effectiveness. This ensures targeted, time-controlled drug delivery. This article emphasizes the development of phytochemical-based nanocarriers corresponding to breast cancer. Moreover, the article presents the unhighlighted parts of the therapeutical industry to help patients. Enhancing patients’ quality of life would uplift the healthcare system.

**Abstract:**

As the world’s most prevalent cancer, breast cancer imposes a significant societal health burden and is among the leading causes of cancer death in women worldwide. Despite the notable improvements in survival in countries with early detection programs, combined with different modes of treatment to eradicate invasive disease, the current chemotherapy regimen faces significant challenges associated with chemotherapy-induced side effects and the development of drug resistance. Therefore, serious concerns regarding current chemotherapeutics are pressuring researchers to develop alternative therapeutics with better efficacy and safety. Due to their extremely biocompatible nature and efficient destruction of cancer cells via numerous mechanisms, phytochemicals have emerged as one of the attractive alternative therapies for chemotherapeutics to treat breast cancer. Additionally, phytofabricated nanocarriers, whether used alone or in conjunction with other loaded phytotherapeutics or chemotherapeutics, showed promising results in treating breast cancer. In the current review, we emphasize the anticancer activity of phytochemical-instigated nanocarriers and phytochemical-loaded nanocarriers against breast cancer both in vitro and in vivo. Since diverse mechanisms are implicated in the anticancer activity of phytochemicals, a strong emphasis is placed on the anticancer pathways underlying their action. Furthermore, we discuss the selective targeted delivery of phytofabricated nanocarriers to cancer cells and consider research gaps, recent developments, and the druggability of phytoceuticals. Combining phytochemical and chemotherapeutic agents with nanotechnology might have far-reaching impacts in the future.

## 1. Introduction

One of the leading causes of fatality worldwide and a major barrier to extending life span is cancer, with breast cancer being among the most prevalent malignancies impacting women worldwide [1]. Women can develop breast cancer at any age after puberty, but the risk increases with age. According to the WHO, 2.3 million women worldwide had breast cancer in 2020, and 685,000 of them passed away from it [1]. Despite the notable improvements in survival in countries with early detection programs combined with the broad availability of different treatments, breast cancer continues to represent a significant societal health burden and has a large impact on the global number of cancer deaths due to the rapidly increasing rate of global aging [2]. According to a recent study, the number of new instances of breast cancer will reach more than 3 million cases annually by 2040 (an increase of 40%), and the number of deaths will reach more than 1 million cases annually (an increase of 50%) [3]. Currently, somewhere in the world, a woman is diagnosed with breast cancer every 14 s [4]. Various phytomedicines and nanotechnology-based interventions are under development [1,2,3,4].

**Figure 1:**
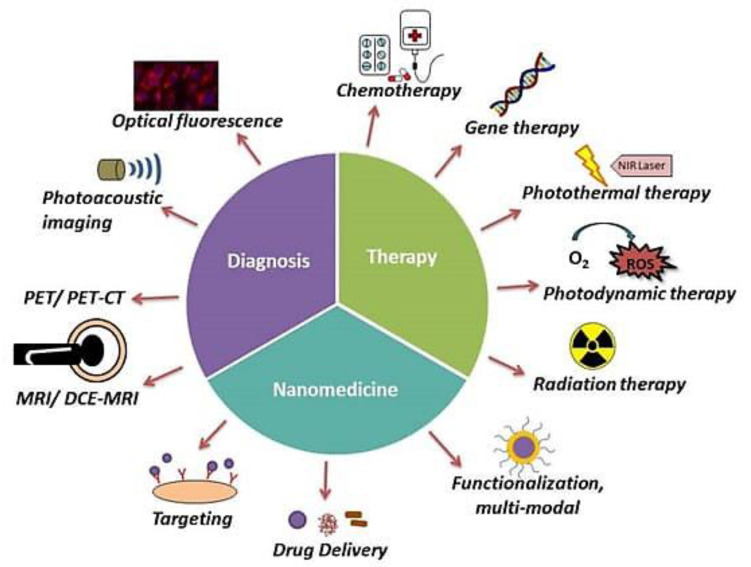
Current State of Breast Cancer Diagnosis, Treatment, and Theranostics. Adapted from [13] under CC BY license.

Breast cancer, which originates from the epithelium of the milk ducts, is a highly heterogeneous neoplasm. It varies within each individual tumor, i.e., intratumor heterogeneity, and it significantly varies between patients, i.e., intertumor heterogeneity [5,6]. The histopathologic categorization of breast cancer is based on intertumor heterogeneity. The most prevalent (40–75%) histologic type of invasive breast cancer is invasive ductal carcinoma. Additionally, there are 21 more specific subtypes with distinct morphologic characteristics included in the WHO classification, among which the most common (5–15%) one is invasive lobular carcinoma [7]. According to the assessment of immunohistochemistry (IHC), the expression of estrogen receptor (ER), progesterone receptor (PR), and human epidermal growth factor receptor 2 (HER2) was found to be 80%, 60–70%, and 15–20%, respectively, in all invasive breast carcinomas [8,9,10]. Breast cancer is divided into four main intrinsic molecular subgroups with therapeutic and prognostic implications based on gene expression analysis: luminal A, luminal B, HER2-enriched, and basal-like [11]. The luminal A and B subtypes exhibit tumor heterogeneity among ER-positive breast tumors and seem to have higher rates of survival than the HER2-enriched and basal-like subtypes [12]. The HER2-enriched subtype, which comprises the ER^−^/PR^−^/HER2^+^ and ER^+^/PR^+^/HER2^+^ cancers, is characterized by elevated expression of the HER2 and proliferating genes. The basal-like subtype is triple-negative in 70% of cases and enriched for genes expressed in basal epithelial cells [11]. Breast tumors that do not express ER, PR, or HER2 are referred to as “triple-negative” breast carcinomas. Figure 1 represents the current state of the diagnosis, treatment, and theranostics of breast cancer. Breast imaging is frequently employed to assess the quality of breast implants, but it also plays a critical role in the detection, diagnosis, and clinical treatment of breast cancer [13]. Chemotherapy, surgical removal of the cancerous tissue, radiotherapy, immunotherapy, and a combination of any of these treatments have been the traditional methods of cancer treatment. Traditional chemotherapeutics are still the main type of treatment for many cancers that are in the late stages, despite obstacles such as systemic toxicity, limited selectivity, and a range of adverse effects [14]. Cancer treatment frequently involves drugs that specifically target cells that divide rapidly, which causes unwanted side effects on healthy, rapidly dividing cells, including hair follicles and the epithelium of the gastrointestinal tract (GIT). The fact that many cancer cells progressively gain resistance to standard kinds of therapy is also one of the exacerbating factors. The requirement for preoperative (neoadjuvant) systemic therapy is established based on the diagnosis and evaluation of the extent of breast cancer. Management for breast cancer requires targeted medicines that are efficient and have few unwanted side effects.

**Figure 2:**
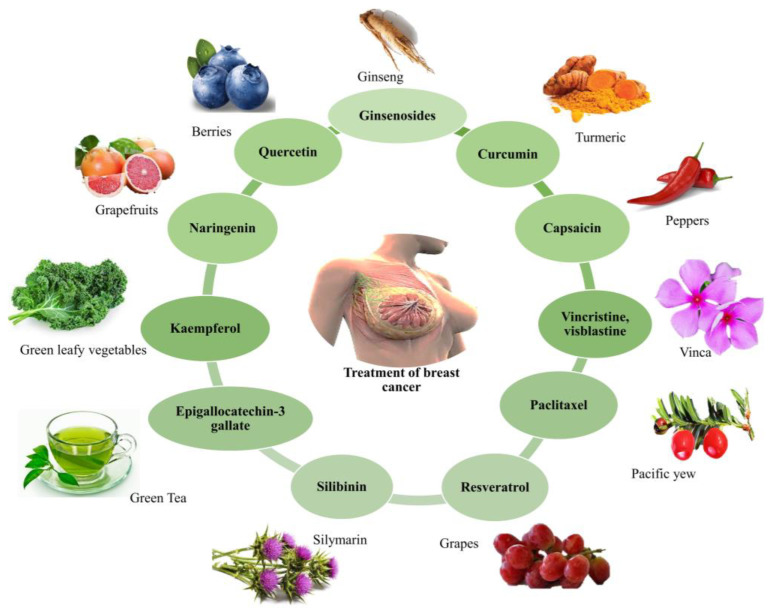
The role of various phytochemicals in the treatment of breast cancer.

Major focus must be placed on reducing global gaps in access to diagnostics, multidisciplinary therapy, and innovative drugs because breast cancer is a global concern. An increasing collection of credible research indicates that phytochemical components taken as nutraceuticals have chemo preventive action on several cancer types [15,16,17]. Figure 2 depicts examples of phytochemicals utilized for breast cancer treatment.

It has been established that phytochemicals, the chemical substances (secondary plant metabolites) produced by plants in their various parts, are ideal candidates for the treatment. Various studies have revealed that such phytochemicals can act as chemo protectants that can control cellular and molecular processes such as DNA repair, apoptosis, cell proliferation, the cell cycle, and metastasis [18,19]. Many of these organic substances are also often comparatively less harmful and better tolerated by healthy cells. This is because many natural products are tolerated by normal cells, even at high dosages compared to chemotherapy medications.

Despite significant work in preclinical settings, there has been little progress in translating phytochemicals to humans [2,19]. One of the many causes of clinical failure may be the ineffective transport of promising natural substances to the target site. Therefore, it is crucial to develop novel efficient delivery methods that can minimize these drawbacks.

The use of nanoparticles (NPs) in medicine has made it possible to create medication delivery methods that are nanoformulated. Common drug carriers include micelles, polymeric dendrimers, quantum dots (QDs), microspheres, nanoemulsions, gold nanoparticles (GNPs), hydrogels, and liposomes. These drug carriers require different techniques for drug attachment, such as encapsulation, covalent binding, and adsorption [20].

Natural agent delivery methods based on nanotechnology have several benefits. One benefit of this nanotechnology is the ability to shield pharmaceuticals enclosed in nanoparticles from the damaging effects of external media, which provide prolonged systemic circulation [21]. Additionally, when compared to non-encapsulated free drugs, nanoparticles can augment the delivery of water-insoluble drugs, improve the passage of chemotherapeutic agents across cell membranes, allow the drugs to only be delivered to cancer cells, enhance drug distribution, offer sustained release of the drug, and assist in the delivery of two or more drugs for combined therapy [22].

### 1.1. Current Limitations of Breast Cancer Chemotherapy Regimens

Chemotherapy medications, which target rapidly proliferating cancer cells, can also harm rapidly proliferating healthy cells, including those in the bone marrow, digestive tract, and hair follicles [23]. However, after the course of treatment is complete or within a year of finishing chemotherapy, these side effects frequently fade away. They might last for a while in some instances. Hair loss, fatigue, loss of appetite, nausea and vomiting, constipation or diarrhea, mouth sores, changes to the skin or nails, neuropathy, chemo brain, and fatigue are among the more frequent short-term adverse effects. Although, infertility, bone thinning, heart damage, leukemia, and other long-term side effects of some chemotherapy medicines for breast cancer are also possible [24].

Due to its drug resistance and tendency to metastasize to distant organs such as the lymph nodes, bone, lung, and liver, breast cancer accounts for the majority of cancer-related fatalities in women [25]. The ATP-binding cassette (ABC) family protein, whose higher expression is correlated with higher resistance to chemotherapy, is widely established to have a significant role in drug resistance in a variety of malignancies. The excessive expression of proteins, such as P-GP1/ABCB1 (P-glycoprotein 1, also known as ATP-binding cassette subfamily B member 1 or ATP-dependent translocase ABCB1) and BCRP/ABCG2 (breast cancer resistance protein, also known as ATP-binding cassette subfamily G member 2 or broad substrate specificity ATP-binding cassette transporter ABCG2), causes multidrug resistance (MDR), which is a significant barrier to the diagnosis and treatment of breast cancer.

It is now recognized that the control of breast cancer and the spread of its metastasis involves a number of routes [26]. Understanding the biological activity of progesterone receptors (PRs), estrogen receptors (ERs), and human epidermal growth factor receptor 2 (also known as receptor tyrosine–protein kinase EGRB-2 or tyrosine kinase-type cell surface receptor HER2) for various subtypes of breast cancer has advanced. Despite recent developments in finding small molecules, proteins, and peptides for immunotherapy, controlled-release drug delivery and targeting are still not possible [27].

To aid in the detection and treatment of breast cancer, nanoparticles (NPs) bearing anticancer medicines can be actively or passively administered to the targeted tumor. NPs have numerous useful characteristics. To test drug effectiveness and overcome MDR, the controlled release of medicinal chemicals from NPs has been accomplished [28,29,30].

### 1.2. The Phytotherapeutics: Benefits and Their Delivery Challenges

Approximately 70% to 80% of the world population prefers herbal therapy as their primary type of treatment, making it one of the most significant forms of traditional medicine [31]. “Phyto pharmaceutical drug refers to an extract of a medicinal plant or a part of it that has been purified and standardized with defined minimum four bioactive or phytochemical compounds, for internal or external use by humans or animals for diagnosis, treatment, mitigation, or prevention of any disease or disorder” [32]. Due to the ineffectiveness of contemporary treatments for chronic diseases and because those treatments rarely show unfavorable serious side effects, the use of herbal medicines has become increasingly widespread in today’s world. Many modern medications and their synthetic analogs have been developed based on the prototype compounds discovered in and isolated from plants. A few examples include vinblastine and vincristine from *Catharanthus roseus*, L-Dopa from *Mucuna prurita*, reserpine from *Rauvolfia serpentine*, and paclitaxel from *Taxus brevifolia* [33].

Notwithstanding the high worldwide breast cancer prevalence, the number of breast cancer patients who employ complementary and alternative therapies (CAMs) in addition to chemotherapy and radiation treatment is rising [34]. CAM is described as a group of methods, systems, and products from the medical and healthcare industries that are typically not included in the scope of mainstream medical care [35]. Herbal remedies or phytotherapy are the most widely utilized and oldest type of CAM practiced on cancer patients [36]. The biological effects of herbal medicines in the treatment of cancer can be wide-ranging and include enhancing the body’s potential to fight cancer by increasing its ability to detoxify or clean itself, changing the way certain hormones and enzymes function, reducing the side effects and complications of chemotherapy and radiation treatment, and enhancing the body’s immune system function, such as enhancing the synthesis of cytokines (interferon, interleukin, colony-stimulating factor, tumor necrosis factor, etc.) [37]. Moreover, it is clear that oxidative stress has a role in the development of cancer and that antioxidants play a role in both cancer prevention and cancer treatment, and the majority of plants are good providers of antioxidants. The majority of malignancies may be related to food, according to numerous research. Furthermore, dietary adjustments can lower the chances of the majority of malignancies [38]. The majority of herbal active ingredients are hydrophobic and have poor solubility. The restricted clinical usage of herbal medications is due to the poor solubility and hydrophobicity of their active ingredients, which results in poorer bioavailability and greater systemic clearance, necessitating repeated administration or an increased dose. Nano or micro formulations, however, can address these issues. Various types of polymer or lipid carriers are found in nanocarriers or sustained-release dosage forms, which are utilized to deliver drugs by a number of routes, such as transdermal, buccal, oral, and parenteral. They aid in greater therapeutic efficacy and localization at the desired target, which increases patient compliance [39]. For instance, oral polymeric nanoparticles can reduce the poor water solubility of *Cuscuta chinensis* [40]. Camptothecin’s poor water solubility and harmful effects can be mitigated by intravenous injection, hydrogel of polymer conjugations, biodegradable implants, liposomes, polymeric nanoparticles, or solid lipid nanoparticles [41,42]. 

Although the use of herbal medicine has dramatically increased recently, there is still a dearth of research data in this area. The greatest hazard to consumer health is expected to result from the quality of herbal medicine being compromised as a result of adulteration and substitution caused by the rising demand for phytopharmaceutical medications on the worldwide market. The main problem for regulatory authorities is finding and identifying high-quality phytopharmaceuticals since interspecies diversity and uncertainty over vernacular nomenclature can lead to the adulteration and misidentification of raw materials for phytopharmaceutical drugs. In this review, we emphasize the recent advancements in understanding the mechanisms of action of phytochemical nanocarriers on various molecular pathways associated with breast cancer. We also discuss the phyto nanocarriers that are in clinical trials and their lacunas for commercialization.

## 2. Advanced Phytochemical Delivery Strategies

**Figure 3:**
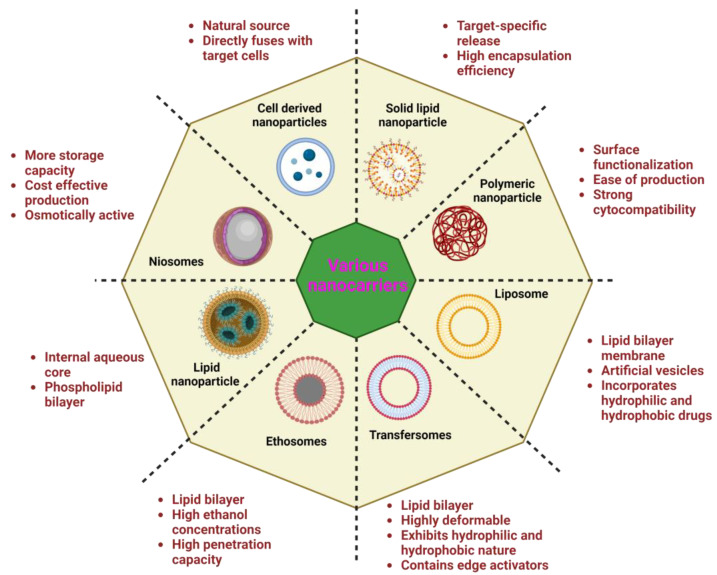
Types of nanocarriers with their characteristics. Created with BioRender.com accessed on 24 January 2023.

Many plant-derived secondary metabolites have low solubility and undesirable stability, restricting their use in therapeutic studies. For example, phenolic phytoconstituents have high antioxidant capability but are unstable under experimental settings. Furthermore, despite their great potential activity, bioactive compounds, such as paclitaxel and curcumin, have limited solubility and bioavailability, necessitating the use of hazardous solvents. The accompanying sections list the existing innovative delivery techniques for loading phytoconstituents for therapeutic effectiveness against breast cancer. Figure 3 depicts various types of nanocarriers with their properties. 

### 2.1. Phytochemical-Loaded Nanocarriers

#### 2.1.1. Polymeric Nanoparticles (PNs)

Polymeric nanoparticles (PNs) have recently emerged as promising nanomaterials because of their desired characteristics, such as simplicity of surface functionalization, ease of production, strong cytocompatibility, and low toxicity. One of the most common methods of tailored drug delivery for treating breast cancer is the incorporation of phytonutrients into PNs. In this system, the phytochemical is either physically encapsulated into or covalently linked to the polymeric matrix.

PNs are globally classified as natural and synthetic PNs. Natural polymers, such as hyaluronic acid, chitosan, alginic acid, heparin, ethyl cellulose, and protein bovine serum albumin (BSA), are used for their excellent encapsulation efficiency and less intrusive nature, and they are strongly recommended. Synthetic polymers such as poly(lactic acid) (PLA), polyglycolic acid (PGA), poly(lactic-co-glycolic acid) (PLGA), poly anhydride (PLA), and poly(sulfobetaine methacrylate) (PSBMA) are also used for the fabrication of PNs. Phytochemical-loaded PNs are manufactured using conventional techniques, such as nanoprecipitation, layer-by-layer assembly, ionic gelation, and emulsion evaporation [43].

**Figure 4:**
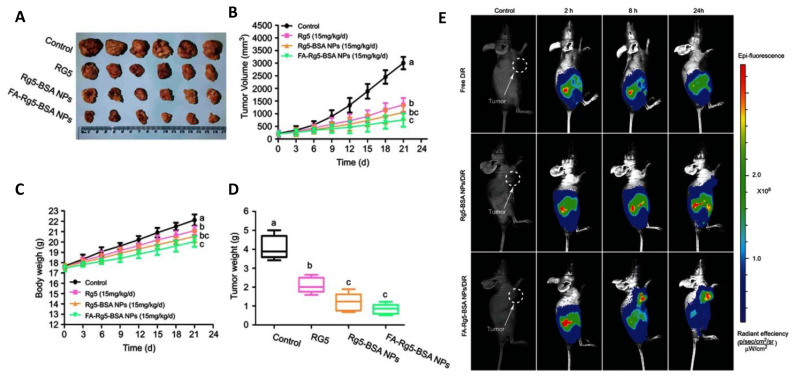
Ginsenoside-fabricated BSA NPs exhibited significant inhibition of breast cancer xenografts (**A**) tumor size, (**B**) tumor volume, (**C**) body weight, (**D**) tumor weight, and (**E**) in vivo bioluminescence after treatment for 21 days. Different letters (a,b,c) indicate significant differences between each group. Adapted with permission from [44] under (CC BY-NC 3.0).

In a recent study, ginsenoside Rg5-loaded BSA NPs were developed using the desolvation process to increase the therapeutic effectiveness and tumor targetability of Rg5. The produced NPs were shown to disintegrate under acidic conditions but had good stability for eight weeks at 4 °C. Additionally, compared to free Rg5, the drug-loaded BSA NPs demonstrated better anticancer efficacy in MCF-7 cells, most likely by facilitating greater absorption of the drug and leading to more efficient cell death induction. Folic-modified drug-loaded BSA NPs outperformed free Rg5 and drug-loaded BSA NPs in an in vivo anticancer study using an MCF-7 xenograft mouse model for suppressing tumor growth. According to the in vivo real-time bioimaging analysis, the produced NPs had a better capacity for tumor accumulation [44] (Figure 4).

Research on polymeric nanoparticles (both synthetic and natural) for administering phyto-derived therapeutic drugs, including curcumin, Epigallocatechin-3-gallate (EGCG), berberine, chrysin, and quercetin, is ongoing on a global scale. In brief, Kumari et al. developed a formulation based on curcumin nanoparticles and PGMD (poly-glycerol-malic acid-dodecanedioic acid) for anticancer efficacy against breast cancer cells. Both nanoparticles had entrapment effectiveness between 78% and 81%. The scratch assay and in vitro anticancer activities were performed on the breast cancer cell lines MCF-7 and MDA-MB-231. In the MCF-7 cell line, the IC50 of the nanoformulation was found to be 40.2 and 33.6 µM at 48 h; in the MDA-MB-231 cell line, it was 43.4 and 30.5 µM. This research revealed that the nanoparticles have more anticancer efficacy than curcumin alone [45]. 

Zeng et al. used nanotechnology that increased the capacity of EGCG to target MCF-7 cells. Two different types of EGCG nanoparticles (FA-NPS-PEG and FA-PEG-NPS) were developed, and their properties and effects on MCF-7 cells were investigated. The findings showed that I FA-NPS-PEG and FA-PEG-NPS both have great stability, and their particle sizes were 185.0 ± 13.5 nm and 142.7 ± 7.2 nm, respectively. Their encapsulation efficiencies of EGCG were 90.36 ± 2.20% and 39.79 ± 7.54%, respectively. EGCG nanoparticles, specifically FA-NPS-PEG and FA-PEG-NPS, have been modified by folic acid and polyethylene glycol. These nanoparticles outperformed EGCG in terms of cellular uptake, the inhibition of MCF-7 cell proliferation, and the modification of the expression of several important PI3K-Akt pathway regulatory proteins [46]. Solanki et al. used the desolvation process to encapsulate berberine in BSA NPs, which are nanoparticles made of bovine serum albumin. For BSA NPs and berberine-BSA NPs, the average particle size of the produced nanoparticles was determined to be 116 and 166 nm, respectively. With a drug loading capacity of 7.78%, produced nanoparticles were shown to have an 85.65% drug entrapment efficiency. The BBR-BSA NPs were more cytotoxic to MDA-MB-231 breast cancer cells, according to an apoptotic and cellular uptake analysis. Still, increased intracellular uptake data suggest that berberine-BSA NPs could significantly boost anticancer activity at a lower dose of berberine [47].

In addition, Sulaiman et al. used a nano participation approach to develop nanochrysin or PLGA-PVA that was loaded with chrysin successfully. The current research demonstrated the cytocompatibility of the modified nanochrysin. The modified nanochrysin’s in vitro anticancer activity toward the MCF-7 and SKOV-3 cell lines was examined. It was observed that the nanochrysin exerted dose-dependent cell growth arrest against both cancer cells. Compared to pure chrysin, the IC_50_ value of nanochrysin was substantially lower and promoted the apoptotic cell death pathway. As shown by the apoptotic assay techniques, it also exhibited anti-oxidant activity, a protective effect against DNA damage under H_2_O_2_ activity. The creation of a drug delivery system for the treatment of various cancers may benefit from the modified nanochrysin’s high encapsulation efficacy, small particle size, and gradual release capabilities [48]. Furthermore, Zhou et al. developed PLGA-TPGS (D-α-tocopherol polyethylene glycol 1000 succinate)-based polymeric nanoparticulate systems for quercetin (Qu-NPs) oral delivery. They assessed the formulation’s anticancer activity against triple-negative breast cancer both in vitro and in vivo. The average diameter of Qu-NPs is 198.4 ± 7.8 nm, with a high drug loading capacity of 8.1 ± 0.4%. The Qu-NPs showed noticeably better inhibition of triple-negative breast cancer cell growth and metastasis. After oral gavage, 4T1-bearing mice showed a strong anticancer response to Qu-NPs, with a tumor inhibition ratio of 67.88% and fewer lung metastatic colonies. Additionally, quercetin’s inhibitory effect on migrating MDA-MB231 cells with uPA knockdown was significantly reduced. Through the combined inhibition of urokinase-type plasminogen activator (uPA), Qu-NPs enhanced the anticancer and anti-metastatic effects that were already present [49].

#### 2.1.2. Cell-Derived Nanovesicles (CDNs)

The ongoing worry about the biosafety of employing synthetic materials to transport natural products has accelerated the discovery and use of cell-derived nanovesicles (CDNs) [50]. Generally, CDNs are isolated from various natural sources (blood, milk, and cell culture media) and natural plants. Differential centrifugation, immunoaffinity separation, gel filtration chromatography, ultrafiltration, and polymer precipitation are used to separate or extract them. CDNs are created by isolation, biofabrication, and biolipid-based reconstruction. It was also shown that the exogenous stimulation (ultra-sonication and extrusion) of host cells increases the yield of CDNs [51,52].

There are two approaches to loading cargo (phytoconstituents) into CDNs: preloading and post-loading. Endogenous preloading is achieved by combining phytochemicals with host cells, after which spontaneously generated CDNs or biofabricated CDNs with bioactive cargo are collected. However, the preloading strategy is limited to CDNs generated from cell cultures or microorganisms, making it impracticable for plasma proteins or food. Post-loading entails the addition of phytochemicals to the preformed CDNs under various conditions. This approach includes passive incubation, sonication, surfactant permeabilization, and freeze—thaw cycles. Passive diffusion is the most widely used and safe technology for efficiently encapsulating bioactive compounds, such as curcumin and paclitaxel. However, it has poor encapsulation effectiveness [52,53].

Although active loading via electroporation improves the loading of nucleic acids and nanoparticles into CDNs, it can promote aggregation, alter the surface charge, induce instability, and potentially denature bioactive CDNs [54]. A recently proposed approach was the fusion of liposomes containing bioactive molecules with isolated CDNs to generate hybrid nanovesicles. By this approach, one can generate CDNs loaded with multiple bioactive molecules.

Furthermore, surface modification of the active target at specific sites can result in the increased targeting ability of CDNs with minimal toxicity and dosage reduction. CDN modification can be achieved either pre- or post-generation. In the first case, the host cells are incubated with phospholipids, such as DSPE-PEG-RGD and hyaluronic acid, resulting in the generation of functionalized CDNs [55]. In contrast, CDNs are enhanced in terms of membrane modification, protein derivatization, and lipid rectification in the post-modification technique [56]. Although active target modification enables specific targeting, the influence of ligand representation on the immune system, long-term stability, and loading capacity must be extensively investigated. Furthermore, CDNs may be functionalized by employing a variety of exogenous (temperature, photo responsive, and NIR) and endogenous (pH, enzyme overexpression) stimuli to create smart CDNs [52]. Bioactive phytochemicals, such as polyphenols, flavonoids, terpenoids, and alkaloids, have been exploited for CDN-based delivery. Table 1 lists the phytochemicals given against breast cancer via CDNs [53].

#### 2.1.3. Lipid Nanoparticles

Lipid-based nanodrug delivery systems are among the most promising colloidal carriers for phytochemicals. Lipid-based nanoparticles, such as liposomes, solid lipid nanoparticles (SLNs), and nanostructured lipid carriers (NLCs), can transport hydrophobic and hydrophilic molecules utilizing the phospholipid bilayer or internal aqueous core.

Liposomes are biocompatible and biodegradable and exhibit quite low toxicity. In addition, integrating hydrophilic and lipophilic drugs, developing lipid domains, fluidity, polyvalent binding, fusion, longer retention of drugs, high drug loading capacity, site-specific targeting, and controlled drug release are further benefits of liposomes. Furthermore, they provide prolonged restorative effects, a reduced likelihood of probable adverse effects, and enhanced protection for drugs against physiological conditions [63]. Therefore, it is considered a smarter choice to incorporate anticancer phytochemicals into liposomes to overcome the constraints of the inability of traditional chemotherapy to suppress carcinogenesis and their ability to reduce lethal side effects completely.

**Figure 5:**
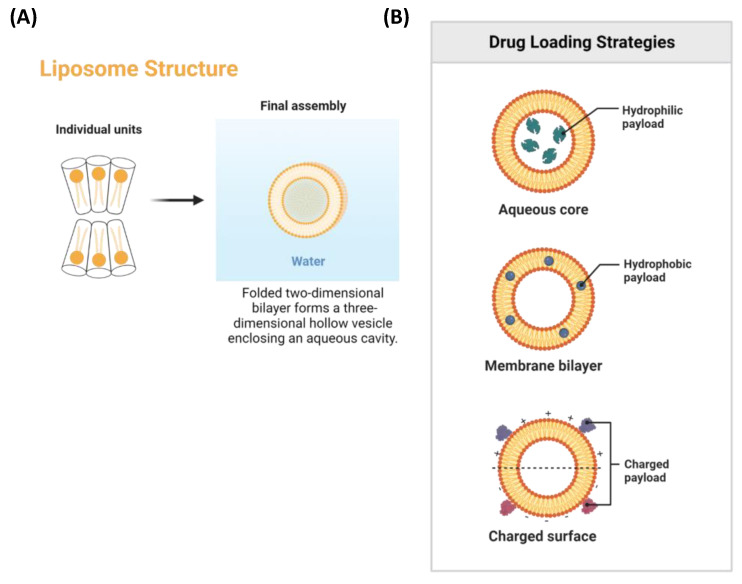
(**A**) Liposomal structure, (**B**) Various drug loading strategies with liposomes. Created with BioRender.com accessed on 24 January 2023.

These liposomes have a spherical shape, are composed of nontoxic phospholipids and cholesterol, and are surrounded by water (Figure 5A). They create vesicles in the presence of an aqueous solution that increases the stability and solubility of anticancer drugs while encapsulating them in liposomes. Various payloads with hydrophobic and hydrophilic molecules and charged molecules can be incorporated into liposomes (Figure 5B).

In recent years, formulations of liposome-based phytochemicals have gained popularity. Chitosan and lecithin were used by Deshmukh et al. to create a liposomal nanosystem by an electrostatic deposition-assisted film hydration approach to safeguard chrysin (also known as 5,7-dihydroxyflavone, a flavone found in honey, propolis, passion flowers, *Passiflora caerulea* and *Passiflora incarnata*, and *Oroxylum indicum*) in the nanolipoidal shell [64]. Chrysin was encapsulated into liposomes and demonstrated increased anticancer efficiency in the MCF-7 cell line. After encapsulation in a liposome, chrysin’s relative bioavailability increased five-fold, according to an in vivo pharmacokinetic study [64]. Quercetin and mycophenolic acid-loaded liposomes were independently synthesized by Patel et al., and both in vitro and in vivo experiments were then carried out [65]. Another study used a thin-layer hydration approach to develop thermosensitive betulinic acid-loaded magnetoliposomes. This study showed that MDA-MB-231 breast cancer cell lines and MCF 10A nontumor cell lines were more susceptible to the heightened effects of hyperthermia, as shown by in vitro experiments using the MTT assay, scratch assay, and LDH assay [66]. Furthermore, an in ovo study depicted the antiangiogenic effect of Lip + MIONPs + BA during hyperthermia treatment. In addition, resveratrol-containing liposomes that were coated with peptide and sucrose were used to treat breast cancer [67]. In vivo investigations on mice with breast cancer showed greater efficacy at lower dosages when compared to free resveratrol, with an IC_50_ of 20.89 mol^−1^ in MCF-7 breast cancer cell lines. The authors also demonstrated the downregulation of B-cell lymphoma-2 cells by increasing the expression of p53 [67]. Furthermore, pegylated liposomes were formulated with the combination of docetaxel and its 3-N-pentadecylphenol derivative. Due to the pegylated hydrogenated soy PC-3-N-pentadecylphenol derivative, docetaxel demonstrated improved stability over 30 days. MDA-MB-231 and MCF-7 breast cancer cell lines showed strong cytotoxic effects [68].

On the other hand, SLNs are a new class of safer and more effective gene/drug delivery systems. SLNs, which range in size from 50 nm to 1 µm, are submicron colloidal carriers made of physiological lipids distributed in an aqueous solution, and these physiological lipids remain solid at body and room temperature. SLNs provide several fascinating benefits, including physical stability, increased drug selectivity, protection of absorbed compounds from clearance and degradation, avoidance of organic solvent residue, simplicity in manufacture, low cost, and nontoxicity [69,70]. Therefore, SLNs have been widely employed to deliver phytochemicals, including curcumin, berberine, resveratrol, quercetin, and EGCG [70,71,72,73,74], to increase anticancer activities, protect labile molecules, increase oral bioavailability, and reduce adverse effects.

#### 2.1.4. Transferosomes

Transfersomes are novel drug delivery systems that consist of phospholipids and a membrane-softening agent (such as Tween 80, Span 80, and sodium cholate), acting as edge activators (EAs) that facilitate the ultra deformable property of the transfersomes [75]. Due to their highly elastic nature, transfersomes can deform and squeeze themselves as intact vesicles through narrow pores, which are noticeably smaller than the diameter of transfersomes [75]. Transfersomes can pass through the intact stratum corneum along two intracellular lipid pathways distinct from one another in terms of their bilayer characteristics. High deformability and both hydrophilic and hydrophobic characteristics in transferosomes allow for improved intact vesicle penetration. This system is significantly more elastic and flexible than liposomal drug delivery, favoring effective skin penetration and, by extension, acting as an innovative drug delivery system [76]. Transferosomes avoid the obstacle of skin penetration by squeezing along the intracellular sealing lipid of the stratum corneum [77]. The transfersome membrane is made flexible by combining a suitable surface-active agent in appropriate proportions [78]. In general, anionic surfactants are typically more successful at improving skin penetration than cationic surfactants, and the critical micelle concentration is likewise lower.

Nonionic surfactants with an uncharged polar head group are more tolerable than cationic and anionic surfactants [79]. Nonionic surfactants are thought to be less harmful, less hemolytic, and less irritating to cellular surfaces. They also tend to keep the pH of a solution close to physiological levels. Additionally, they serve various purposes, including their roles as solubilizing, emulsifying, and potent P-glycoprotein inhibitors, which are beneficial for increasing drug absorption and targeting particular tissues [80]. Transferosome delivery systems assure optimal distribution, increased bioavailability, and promising phytoactivity stability in herbal formulations [81]. These systems offer numerous advantages that include the accommodation of pharmaceuticals with various solubilities due to their hydrophobic and hydrophilic moieties, high entrapment efficiency, deformation and narrow pass-through constriction, biocompatibility and systemic as well as topical delivery of the drug [82,83,84,85].

Gadag et al. demonstrated the possibility of transpapillary transfer of resveratrol, a phytochemical, for treating breast cancer. In this study, the biomaterial soy phosphatidylcholine was used to encapsulate resveratrol into transfersomes (RVT-TRF) to provide sustained release of the drug. Iontophoresis accelerated RVT-TRF passage through the mammary papilla and into the breast tissue. In vitro transpapillary iontophoresis investigation on porcine mammary papilla revealed that RVT-TRF penetrated more readily than passive diffusion. Further evidence for transpapillary transport came from an in vitro fluorescence microscopy experiment with fluorescein-conjugated RVT-TRF. Compared to the oral administration of pure resveratrol, the optimized RVT-TRF demonstrated a greater maximum peak plasma concentration (C_max_) and area under the curve (AUC) [86].

#### 2.1.5. Ethosomes

Ethosomes are vesicles made of phospholipids, a high proportion of ethanol (20–50%), and water. The high ethanol concentrations in ethosomes alter the skin’s lipid bilayer and increase the vesicles’ capacity to penetrate the stratum corneum [87]. In terms of the proportion of ethanol, vesicular bilayer fluidity, mechanism of permeation through the skin, methods of preparation, and lack of adverse effects, ethosomes stand out from other lipid nanocarriers. The distribution of therapeutic drugs into a deeper epidermal layer and systemic circulation is made easy and successful by ethanol’s efficient penetration enhancer function. Numerous compounds, including hydrophilic, lipophilic, and high molecular weight drugs, can be encapsulated by ethosomes [88]. Both occlusive and nonocclusive situations allow ethosomes to transfer the drugs over the skin successfully [89,90].

Ethosomes are vesicles that range in size from 30 nm to several microns. They are soft and flexible. It has been noted that when made using the same approach without any size-reduction steps, the size decreases with an increase in ethanol concentration from 20 to 45% and is caused by the high alcohol content. The vesicles obtain a net negative charge from ethanol, reducing their size [91,92]. Nasr et al. encapsulated thymoquinone (TQ), the main biologically active complex of black cumin seeds, in ethosomes by the response surface method. They applied it to in vitro breast cancer potential assessment. In this study, toxicity and release curves were established, and ethosomic TQ had higher cytotoxic activity than free TQ against MCF-7 cell lines. Free TQ and ethosomic TQ were found to have IC_50_ values of 1.10 µg/mL and 0.95 µg/mL, respectively [93].

#### 2.1.6. Niosomes

Niosomes are novel drug delivery systems, nanometric lamellar vesicles created when a nonionic surfactant is combined with a cholesterol-like helper lipid. The nonionic surfactants use energy to produce a stable bilayer vesicle in hydrophilic situations (physical agitation and heating). While the hydrophilic heads in the bilayer structure remain in contact with the aqueous side, the hydrophobic sections are directed away from it. Niosome preparation requires the use of surfactants that are biocompatible, biodegradable, and nonimmunogenic. In vivo and in vitro, niosomes function similarly to liposomes by extending the circulation of the phytochemical that is encapsulated, modifying its organ distribution and enhancing bioavailability. Niosomes can improve the solubility and stability of phytochemicals, and targeting and regulating phytochemical release is their intended function.

Barani et al. developed niosomes of two distinct formulations that contained thymoquinone (TQ, a phytochemical compound found in *Carum carvil* seeds) and *C. carvil* extract (Carum) (Nio/TQ and Nio/Carum, respectively) and the properties of the resulting niosomes were investigated [94]. Compared to free TQ, both loaded formulations offered regulated release. The MTT assay demonstrated that loaded niosomes have more anticancer activity against the MCF-7 cancer cell line than free TQ and Carum. These findings were supported by a flow cytometric study. Cell cycle analysis revealed G2/M arrest in the formulations of TQ, Nio/TQ, and Carum. TQ, Nio/TQ, and Nio/Carum all significantly reduced the migration of MCF7 cells. These findings indicate that novel carriers with great effectiveness for encapsulating low soluble phytochemicals include TQ and Carum-loaded niosomes, which would also be advantageous systems for treating breast cancer [94].

In another study, neosomes containing Lawsone (2-hydroxy-1,4-naphthoquinone, also known as hennotannic acid, a major constituent of the henna plant (*Lawsonia inermis*)), nonionic surfactants, and cholesterol were prepared. An in vitro study showed that encapsulating Lawsone in niosomes significantly increased the anticancer activity of the formulation in the MCF-7 breast cancer cell line compared to the free Lawsone solution [95].

Recently, folate-targeted curcumin-loaded niosomes for site-specific delivery in breast cancer were investigated [96]. This study used folic acid (FA) and polyethylene glycol (PEG) to decorate synthesized curcumin-loaded niosomes to prevent breast cancer. Compared to the free drug and developed niosomes, it showed a significant increase in Bax and p53 gene expression levels. Bcl2 levels were lower with PEG-FA decorated niosomes than with undecorated niosomes and the free drug. The PEG-FA-modified niosomes showed the most preponderant endocytosis in the MCF7 and 4T1 cell uptake assays. The produced nanoformulations were taken up by breast cancer cells and sustained drug-release properties [96].

### 2.2. Phytochemical-Assisted Nanocarriers

Significant effort has recently been made toward synthesizing metal nanoparticles utilizing plant extracts as reducing agents to adhere to the general principles of green chemistry [97,98]. These methods have been shown to be economical and environmentally friendly ways to create different metal nanocomposites. Alkaloids, flavonoids, terpenoids, soluble carbohydrates, phenolic acids, and alkaloids are a few phytochemicals found in plants. They can act as reducing and stabilizing agents in the production of metal nanoparticles. Hence, this phytochemical-assisted synthesis of nanoparticles is a very promising technique for synthesizing nanoparticles, as the plant itself serves as a capping and reducing agent. Both intracellular and extracellular nanoparticle synthesis is possible in plant systems [99]. Growing the plant in organic media containing metal-rich elements, metal-rich soil, or metal-rich hydroponic solution are all examples of intracellular strategies for nanoparticle synthesis [100,101,102]. In addition, extracellular approaches use leaf extracts made by boiling and crushing leaves to create nanoparticles [103].

On the other hand, plant-derived edible NPs exhibit an economic advantage in scaling up for mass production [104]. The main obstacles between the laboratory and the clinic in nanomedicine, as is widely known, are biocompatibility and safety. Due to their high quantities of lipids, low levels of proteins, and abundance of RNAs, plant-derived edible NPs have a distinct advantage in these areas and are among the safest therapeutic NPs [105]. The formation of tumors in the leukemia mouse model is effectively inhibited by edible NPs derived from Citrus limon. It should be mentioned that extracting edible NPs with high yield and quality is challenging. The use of isosmotic buoyant density and isosmotic cushion ultracentrifugation, equilibrium density gradient ultracentrifugation, and differential ultracentrifugation combined with density gradient centrifugation have all recently emerged as promising extraction and purification methods [106]. In addition to their natural ingredients, edible plant-derived lipid nanoparticles can be employed as nanocarriers for chemical drugs. Phytochemicals or chemotherapeutic drugs can be encapsulated in nanostructures created from plant lipids using sonication [107]. Lipid nanoparticles, a unique and organic delivery technology, are easily biodegradable and free of environmental biohazards. These plant-derived lipid nanoparticles offer drug delivery to a particular site of the human body [108].

## 3. Evidence of the Role of Phytofabricated Nanocarriers against Breast Cancer

### 3.1. Anticancer Activity

#### 3.1.1. Immunostimulation

Sijia et al. synthesized tea nanoparticles (TNPs) loaded with doxorubicin (DOX), and their in vitro immunostimulatory and anticancer activities were studied [109]. The TNPs significantly increased IL-6, TNF-α, and G-CSF in RAW264.7 macrophages and exhibited the ability to modulate macrophage immunostimulation. In addition, in comparison with free DOX, the DOX-loaded TNPs facilitated the intracellular delivery of DOX in sensitive (MCF-7) and resistant breast cancer cells (MCF-7/ADR) with enhanced in vitro cytotoxicity of IC50 (MCF-7-0.036 ± 0.012 and MCF-7/ADR-15.16 ± 7.05). The formulation exhibited pH-responsive release of doxorubicin, favoring in vivo antitumor applications [109]. Despite having an anticancer impact, there are only a few in vivo cancer investigations using TNPs, since it is unclear how exactly their toxicity is induced. TNPs may have several therapeutic benefits in cancer therapy if research continues to improve in this direction.

#### 3.1.2. Apoptosis

The antitumor effects of quercetin have been thoroughly studied in a wide range of malignancies. Their potential was explored by Minaei et al. in the fabrication of mixed NPs by combining quercetin and lecithin for doxorubicin-induced apoptosis. The results of this investigation showed that the addition of nano-quercetin to doxorubicin enhanced its toxicity in the MCF-7 cell line [110]. In addition, a study from Kazi et al. with folate-decorated epigallocatechin-3-gallate loaded PLGA nanoparticles (FP-EGCG-NPs) evaluated the efficacy in breast cancer cells. Treatment with FP-EGCG-NPs in MDA-MB-231 and MCF-7 cells significantly induced cytotoxicity, high apoptotic potential, and high mitochondrial depolarization compared with EGCG alone. Furthermore, in a scintigraphic imaging study, the FP-EGCG-NPs labeled with technetium-99m (^99m^Tc, a metastable nuclear isomer of technetium-99) exhibited tumor selectivity in MDA-MB-231 tumor-bearing nude mice. The US health agency, the National Institute of Health (NIH), recommended betulinic acid for its cell-specific toxicity for cancer chemotherapy.

Halder et al. synthesized lactoferrin (Lf)-attached betulinic acid nanoparticles (Lf-BAnp) for targeting aggressive triple-negative breast cancer (TNBC) cells by understanding the limiting capability of betulinic acid in terms of solubility and cell uptake. Lf-BAnp exhibits potential inhibitory activity against the proliferation and cell viability with cell cycle arrest [111]. The use of gold nanoparticles was significantly higher in nanotechnology due to their ease of production and biocompatibility with broad biomedical applications. Recent studies suggest that phytochemicals such as withanolide-A and Curcuma wenyujin, as natural active drugs in conjugation with AuNPs, can effectively overcome breast cancer drug resistance [112,113]. Additionally, Ruenraroengsak et al. examined the in vitro chemotherapeutic effectiveness of ZnONPs loaded through a mesoporous silica nanolayer (MSN) against drug-sensitive breast cancer cells (MCF-7: estrogen receptor-positive, CAL51: triple-negative) and their drug-resistant counterparts (MCF-7TX, CALDOX). Gold nanostars were coated with ZnO-MSNs (AuNSs). The mesoporous silica nanolayer (MSN)-ZnO-AuNSs decreased the viability of CAL51/CALDOX cells and MCF7/MCF-7-TX cells. In contrast, MSN-ZnO-AuNSs conjugated with Frizzled-7 (FZD-7) increased the toxicity by three times in resistant MCF-7TX cells.

#### 3.1.3. Metastasis

Although medical advancements have significantly changed how BC patients are managed over the past few decades, metastases continue to be challenging to treat because of their resistance to therapeutic agents, molecular heterogeneity, and physiological barriers at different organ sites [114,115]. Systemic chemotherapy does not account for the enormous variations in tumor microenvironments due to the widely dispersed nature of metastasis [116,117]. Nanoformulations show definite benefits with the advancement of liposome or lipid nanoparticle technology, including improved therapeutic characteristics and pharmacokinetics and decreased drug toxicity. Additionally, they can be made to target cancer cells and the tumor microenvironment concurrently for improved targeting and therapy options.

Breast cancer-related mortality is mostly caused by tumor metastasis, which continues to be the main barrier to effective chemotherapy for the disease. α-Tocopherol polyethylene glycol (TPGS) and phosphatidylcholine (PC) were included in silibinin-loaded lipid nanoparticles (SLNs) developed using a thin-film hydration technique to prevent the metastasis of breast cancer. It was further shown that MDA-MB-231 breast cancer cells successfully absorbed the optimized SLNs, with particle sizes of approximately 45 nm and great serum stability. Notably, the SLNs could efficiently and significantly accumulate within tumor tissues. Through the downregulation of MMP-9 and Snail, SLNs had significantly higher inhibitory effects than free silibinin on the invasion and migration of MDA-MB-231 cells. In addition, in the spontaneous and blood vascular metastasis models, SLN treatment resulted in 67% and 39% less pulmonary metastasis formation than saline treatment, respectively. Additionally, TPGS and phosphatidylcholine-based blank lipid nanoparticles (BLNs) were the first to be discovered to have antimetastatic activity. Furthermore, neither of the mouse models treated with SLNs showed any clear signs of systemic toxicity. SLNs can potentially be a potent, low-toxicity tumor-targeted drug delivery system as a promising preventative therapeutic agent against breast cancer metastasis [118].

#### 3.1.4. Angiogenesis

Cancer is a multifactorial disease influenced by genetic, epigenetic, and environmental factors. The culmination of numerous molecular changes causes the normal biological processes regulating cell proliferation, cell survival, genome stability, energy metabolism, and angiogenesis to become dysregulated. Angiogenesis, the quick rise in blood vessel development, is necessary for the availability of enough oxygen and nutrients for the growth of breast tumors. Like all human tissues, breast cancer cells require continuous hydration and oxygenation through the system’s vascular network of capillaries. The angiogenic factors vascular endothelial growth factor (VEGF) A, B, and C, basic fibroblast growth factor (bFGF)/FGF-2, matrix metalloproteinases (MMPs), and IL-8, which are factors linked to breast cancer, are most frequently produced by adipose tissues [119,120]. Plant species contain ursolic acid (UA), a triterpene with anticancer action that its antiangiogenic properties may bring on. However, owing to its poor bioavailability and low water solubility, UA has a limited range of applications.

Rocha et al. created long-circulating, pH-sensitive liposomes containing ursolic acid to solve this problem (SpHL-UA). The authors used the relative tumor volume, dynamic contrast-enhanced magnetic resonance imaging (DCE-MRI), and histological analysis to study the antiangiogenic effects of free UA and SpHL-UA in mouse brain cancer and human breast tumor models. The actions of UA at different phases of tumor formation and its low toxicity have sparked interest in UA as a cancer therapeutic. To assess the antiangiogenic effect of UA in vivo, UA was liposome-encapsulated (SpHL-UA). The therapy with free UA or SpHL-UA utilizing proven tumor-bearing experimental animal models is also described. SpHL-UA did not show antiangiogenic activity in a gliosarcoma model and seemed to induce an antiangiogenic effect in the human breast tumor model [121].

#### 3.1.5. Inhibition of Cancer Stem Cells

Luminal A is the most common breast cancer diagnosed frequently in female patients. Breast cancer stem cells (BCSCs) are a rare group of cells in breast cancer. They are responsible for aggressiveness, medication resistance, relapse, poor treatment response, and an overall decrease in the survival of these cancers. Enhancing the efficacy of breast cancer treatment requires focusing on BCSCs. In addition to expressing stemness markers, these cells can self-renew. A poor clinical outcome is caused by BCSCs, which play a significant role in developing drug resistance. To effectively prevent and cure breast cancer, researchers are working to identify and eliminate most of the tumor mass together with BCSCs. Because BCSCs have abnormal stemness-related gene expression, including CD44, SOX2, OCT4, c-MYC, KLF4, Nanog, and SALL4, they are crucial in the spread of cancer [122,123]. Of all breast cancer subtypes, triple-negative breast cancer (TNBC) has the highest rates of chemoresistance, metastases, and relapse. TNBC is a malignant condition resulting from a self-renewing cell subpopulation known as cancer stem cells (CSCs). They need to be eliminated because they are important limitations of TNBC treatment. In this regard, piperlongumine (PL) was investigated. It possesses extraordinary anticancer properties, but its application is constrained by poor pharmacokinetics. Therefore, a PLGA-based nanoformulation for PL (PL-NPs) was created to increase its biological activity, and the effects of PL and PL-NPs on CSCs in mammospheres were investigated. According to the findings, PL-NPs are more effectively absorbed by cells in mammospheres than PL. Additionally, this study showed that PL-NPs significantly reduce CSC expression of ALDH, self-renewability, chemoresistance, and EMT in mammospheres.

According to further investigation, the suppression of STAT3 may be the primary mechanism underlying these multimodal effects. This was confirmed when combined treatments with colivelin, a potent synthetic peptide STAT3 activator, revealed that the anti-CSC effects of PL and PL-NPs were reversed. All things considered, the data indicate that PL-NPs exhibit greater CSC suppression through the downregulation of STAT3 and shed light on the creation of PL-based nanomedicine for CSC targeting in TNBC [124].

#### 3.1.6. Anti-Proliferative Activities

Most BC tumors have epithelial cell features and express HER-2 (a member of the epidermal growth factor receptor family) or estrogen receptors. Basal cells, which make up approximately one-fifth of BCs, do not fall under any one category of proliferation regulators. Regardless of the cell type, insulin-like growth factor (IGF) signaling is implicated in most BC cells. Cyclin-dependent kinases are activated by transcriptional and nontranscriptional processes in response to all cell proliferation inducers, leading to irreversible progression to the G1/S phase transition. A promising therapeutic approach that first concentrated on the metastatic disease was to disrupt this process. Since most phytochemicals have mechanisms that successfully reduce angiogenesis and cell proliferation, they are viewed as potential anticancer agents. By altering the Wnt/-catenin, PI3K/Akt/mTOR, and MAPK/ERK pathways, quercetin causes cell cycle arrest, which prevents cell proliferation, promotes apoptosis, affects autophagy, and decreases angiogenesis and metastasis in cancer cells [125,126]. In addition, EGCG, a polyphenolic flavonoid produced from green tea, suppresses cancer cell growth, angiogenesis, and migration while causing cell cycle arrest and apoptosis [127].

Furthermore, a limonoid triterpene called nimbolide is generated from Azadirachta indica leaves and flowers. Recent studies have shown that nimbolide inhibits proliferation by downregulating PI3K/AKT/mTOR and ERK signaling, induces ROS-mediated apoptosis and inhibits EMT, migration, and invasion in a variety of solid tumors, including pancreatic, breast, oral, and non-small cell lung cancer, in both in vitro and in vivo systems [128,129,130]. Balakrishnan et al. demonstrated that gold nanoparticle-conjugated quercetin inhibits epithelial–mesenchymal transition, angiogenesis, and invasiveness via the EGFR/VEGFR-2-mediated pathway in breast cancer. In response to AuNP-Qu-5 treatment, a significant decrease in the protein expression of vimentin, N-cadherin, Snail, Slug, Twist, MMP-2, MMP-9, p-EGFR, VEGFR-2, p-PI3K, Akt, and pGSK3 and an increase in the protein expression of E-cadherin were observed. Compared to free quercetin, AuNPs-Qu-5 prevented MCF-7 and MDA-MB-231 cells from migrating and invading. Human umbilical vein endothelial cells (HUVECs) treated with AuNPs-Qu-5 produced fewer capillary-like tubes and had worse cell survival. AuNPs-Qu-5 inhibited the creation of new blood vessels and tubes, according to in vitro and in vitro angiogenesis experiments. DMBA-induced mammary cancer in SD rats was treated with AuNPs-Qu-5, which inhibited tumor growth compared to free quercetin [131].

**Figure 6:**
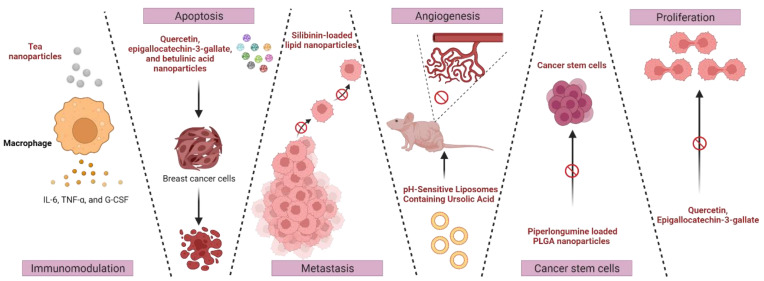
Illustration of the effect of phytofabricated nanocarriers on various mechanisms involved in the progression of cancer. Created with BioRender.com accessed on 21 December 2022.

Various mechanisms of action of phytofabricated nanocarriers on breast cancer discussed in this article are depicted in Figure 6.

### 3.2. Theranostic Targeting

Theranostic approaches are accepted in the treatment of various diseases, including cancer, and incorporate both therapeutic and diagnostic approaches. BC is not overlooked, yet there are very limited studies and current therapeutic applications. Various nanoparticles (NPs), such as gold (Au), silver (Ag), metallic NPs, carbon-containing NPs, and polymers [132], are used in theranostic approaches, and with increasing technology, green synthesis is also justified [132]. Au NPs, when synthesized by chemical or physical means, tend to hold on to toxicity and eliminate excessive heat [133]. When derived from biological sources such as Olax scandens, it produces bearable toxicity, sufficient stability, and reduced immunogenicity [132]. Along with the therapeutic approach, it shows bright red fluorescence, providing an exact location of the MCF7 cell line (breast cell line) [134]. A phytochemical obtained from *Auxemma oncoclyx* named oncocalyxone has potent anticancer efficacy against MCF7 cells [135]. An in vitro study of sesamin showed its efficacy against BC by altering the G1 phase of cell division along with a reduction in cyclin D1, while sitosterol targets the G2/M phase to attain a similar response [136].

Anacardic acid (AA) helps to arrest the cells in the cell cycle (G0/G1) and hence leads to apoptosis [137]. Kushwar et al. stated that when AA is associated covalently with docetaxel (DTX), bovine serum albumin and gemcitabine enhance apoptosis activity and enhance pharmacokinetics [138]. The therapeutic activity on MCF7 cells was higher than that of individual drugs used alone (AA and DTX) [138]. Emodin acts on 4TI breast tumors (a cell line) and suppresses macrophage infiltration, which is complementary to decreased tumor angiogenesis and enhanced T-cell activation [139]. Bimetallic nanostructures are receiving more attention in the developmental phase. A combination of Au–Ag bimetallic NPs proves itself as a better targeting entity [140]. Wu et al. concluded that this hybrid complex structure has a better tendency to absorb near-IR light and hence perform phototherapy in MCF-1 cells while leaving the surrounding cell undisturbed [141].

#### Theranostic-Related Patents

BC is a well-studied disease, yet extra efforts are needed to grasp its maximum effectiveness against this deadly disease. Scientists are attempting to develop innovative management techniques. A patent is a crucial human right provided to the inventor to enjoy the perks of his/her invention. To date, a total of 1,82,000 patents have been published on the topic “Breast Cancer”. The patents filed on BC associated with theranostic approaches are described in Table 2.

## 4. Phytonanomedicines Approved by the FDA or in Preclinical and Clinical Trials

Breast cancer (BC) is the most prevailing cancer in women, and its prognosis has improved over the past few years. The mainstay of BC treatment is still chemoradiation therapy in the early and advanced stages [142]. However, poor selectivity, higher grades of systemic toxicities, and treatment resistance remain the major causes of therapeutic failure among these BC patients [143]. Novel drug delivery systems and drug combinations need to be designed to overcome these problems. Because of the inherent antineoplastic activity of numerous phytochemical components that are bioactive compounds obtained from natural fruits and vegetables, they can be incorporated into the management of various malignant conditions. However, the therapeutic potential of these phytochemical compounds is often hindered because of their poor pharmacokinetic parameters, such as poor solubility, stability, absorption, and quick metabolism. These constituents can be incorporated via nanocarriers, which help enhance their solubility and stability, to address these problems [21,143].

Doxorubicin is a conventionally used molecule for the management of breast cancer; however, it also produces reactive oxygen species (ROS). These ROS damage the different layers of the heart and are responsible for doxorubicin-mediated cardiotoxicity. This issue can be addressed by combining doxorubicin with quercetin, which is a plant-based flavonoid and has good antioxidant potential. The combination of quercetin–doxorubicin decreases the major adverse effect of cardiotoxicity mediated by doxorubicin, which has been observed in numerous in vitro studies. The results of numerous in vivo and in vitro studies show that quercetin dephosphorylates proto-oncogene tyrosine–protein kinase activity and inhibits inflammatory responses in cardiomyocytes. Thus, it protects cardiac myocytes against doxorubicin-mediated cardiotoxicity [144].

Another phytochemical constituent, 6-gingerol, along with paclitaxel, has been tested in vivo and in vitro for breast cancer treatment. Paclitaxel has numerous toxicities at its optimal dose, and therefore, its combination with 6-gingerol enhances its effectiveness at a lower dose. The combination of 5 nM paclitaxel with 10 µM 6-gingerol revealed the same viability as monotherapy with 20 nM paclitaxel [145].

The related in vivo, in vitro, and clinical studies have been mentioned in Table 3 and Table 4 for the treatment of breast cancer.

## 5. Lacunas of Phytofabricated Nanocarriers

Evidence is accumulating in support of an important notion that nanotechnology in general and phytofabricated nanocarriers, in particular, may represent an important solution for many existing challenges related to current breast cancer therapies. In fact, such phytofabricated nanoparticles, being advanced biomaterials characterized by the controllable and stimuli-responsive release of therapeutic agents, favorable biodistribution, great biocompatibility, excellent structural stability in serum, low level of side-effects, and prolonged half-life, clearly represent an exceptional way to noticeably increase the therapeutic efficiency combined with the considerable decrease in the potential toxic side-effects. Different carcinogenic metal ions can be reduced to nanoparticles via the natural antioxidant action of various phytoconstituents (primary and secondary metabolites), such as alkaloids, amino acids, flavonoids, polyphenols, proteins, sugars, tannic acids, and terpenoids [152]. The existing literature indicates that various metal nanoparticles with anticancer properties can be phytogenerated using different plants. Examples include green-synthesized silver nanoparticles (AgNPs) using Artemisia tournefortiana Rchb ethanol extract [153], Morus alba leaf extract [154], Annona muricata aqueous leaf extract [155], *Carissa carandas* aqueous extract [156], Leucophyllum frutescens and *Russelia equisetiformis* extracts [157], aqueous extracts of Acacia arabica (Arabic Gum) and *Opophytum forsskalii* (Samh) seeds [158], *Typha azerbaijanensis* aerial part and root extracts [159], and *Rubia cordifolia* L. leaf extract [160], as well as *Papaver somniferum* L. mediated green synthesis of lead oxide (PbO) and iron oxide (Fe_2_O_3_) nanoparticles [161] and alpha hematite nanoparticle (α-Fe_2_O_3_) production using *Rhus punjabensis* extracts [162], garlic extracts [163], extracts of the *Rheum emodi* root [164], and Salvadora persica aqueous extract [165]. Furthermore, tunable cobalt oxide nanoparticles (CoONPs) were generated using the phytochemicals present in the *Rhamnus virgata* leaf extract [166]; gold nanoparticles (AuNPs) were phytosynthesized using an aqueous extract of Ziziphus spina-christi leaves [167]; selenium nanoparticles (SeNPs) were phytofabricated from the *Carica papaya* extract [168] or using *Portulaca oleracea*-based green synthesis [169]; and gold (Au), iron (Fe), and selenium (Se) nanoparticles were fabricated using various natural plant extracts from the Fertile Crescent, where *Ephedra alata* and *Pistacia lentiscus* extracts were used to synthesize the Au-NPs, and the Fe-NPs and Se-NPs were generated using fruit, peel, and seed extracts of Punica granatum [4].

The listed examples, which likely represent the tip of the iceberg, clearly show that there are multiple options for the phytofabrication of different metal nanoparticles with anticancer properties. It is obvious that with so many possibilities for the phytoproduction of a variety of nanocarriers, one has a broad choice of both anticancer nanoparticles and means for their production. However, multiple questions need to be answered before moving into the commercial use of phytofabricated nanocarriers. Since the same metal nanoparticles can be phytofabricated using different extracts from different parts of different plants, careful comparative analysis of their therapeutic potential, lifetime and structural stability in serum, biodistribution, biocompatibility, and potential toxic side effects should be conducted to select the most promising candidate for commercialization. Among the various factors that must be taken into account at this stage, one should pay very serious attention to the global availability of the plants that are planned to be used for the phytofabrication of the nanoparticles. If the optimal plant is not naturally present at the required quantities in the wild, its plantation should be planned, which obviously will contribute to the cost of phytofabrication. Furthermore, facilities and protocols, which will be utilized for the phytofabrication of nanoparticles, should have a flexible design to allow for a rapid switch between different sources, if needed.

Therefore, although in comparison with traditional technologies for the synthesis of nanoparticles, green synthesis seems to be essentially more economical, the commercial viability of the processes for the mass production of phytofabricated nanocarriers requires careful evaluation.

## 6. Conclusions

Breast cancer is primarily treated by chemotherapy, radiotherapy, and surgical resection; however, the survival rate is still low because of adverse drug reactions, drug resistance, and tumor metastasis. As described in this review, an increasing amount of research has demonstrated the anti-tumorigenic effect of phytochemicals that can modulate cellular events and molecular pathways. However, their pharmacological capability is impeded by their low stability, low water solubility, poor absorption, and rapid metabolism. Nanotechnology-based approaches have shed some light on maximizing the potential use of phytochemicals to overcome formulation challenges. Nanocarriers can enhance the solubility and stability of phytochemicals. Apart from improving solubility and stability, nanocarriers could prolong their half-life and even accomplish site-targeting delivery.

However, the questions of nanotechnology are not yet fully answered in the case of real clinical translation. One of the major shortcomings is that, in general, these PNs can only encapsulate small amounts of actives. Tailor-made nanocarriers conjugated with specific ligands could enable loaded phytoconstituents to function at minimal doses. However, the manufacturing of functionalized nanomedicinal formulations for commercialization is a major obstacle. These shortcomings need to be technologically addressed to maximize the anticancer potential of natural medicines. In this sense, nanotechnology has emerged as a promising drug delivery system strategy in the long run.

## Figures and Tables

**Table 1:** List of the reported CDNs with phytochemicals for breast cancer treatment.

Cargo Loaded	CDNs Source	Preparation	Therapeutic Effect	References
Cucurbitacin B	MDA-MB-231 cells	Isolation, Bio fabrication	Metastasis inhibition	[57]
Paclitaxel	MDA-MB-231 cells	Isolation, Bio fabrication	Excellent antitumor activity	[58]
Withaferin A, anthocyanidins, and curcumin	Milk from Holstein and Jersey cows	Mixing	Inhibits inflammation	[59]
Black bean-derived phytoconstituents	Human mammary (MCF7), prostate (PC3), colon (Caco2), and liver (HepG2) cells	Electroporation	Induces cell death and cell cycle arrest	[60]
Berry-derived anthocyanidins	Raw milk from pasteurized Jersey cows	Simple mixing	Inhibits proliferation and inflammation	[61]
Honokiol (extracted from Magnolia plant)	Mesenchymal stem cells	Sonication	Inhibits cell cycle arrest and apoptosis	[62]

**Table 2:** Patents dedicated to theranostic approaches in breast cancer.

Sr. No.	Patent	Nanoparticle	Remarks	Inventor(s)
1	US10201622B2	Magnetic core Gd-chelates	Target-Matrix metalloproteinases 14 (MMP-14) Imaging-MRI	Paul Loadman, Robert Falconer, Jason Gill, Jianghong Rao, Heike E. Daldrup-Link
2	WO2015014756A1	Magnetic core Gd-chelates	Target-Matrix metalloproteinases 14 (MMP-14) Imaging-MRI	Paul Loadman, Robert Falconer, Jason Gill, Jianghong Rao, Heike E. Daldrup-Link
3	CN104225595A	Aptamer (Cell SELEX)	Target-MDA-MB-231 breast cancer cell Imaging-near Infrared	Ju Yu Xiantian Jiang Wei Ding Lin Yu Junsheng Shen Zhen
4	US20150160222A1	Not clarified	Target-SET/KifC1	Ritu Aneja, Padmashree C.G. Rida
5	US9675714B1	Chitosan functionalized 2D graphene sheets Superparamagnetic iron oxide	Imaging-Nuclear magnetic resonance (NMR)	Subhra Mohapatra, Chunyan Wang
6	US20130323165A1	Magnetic cationic liposomal nanoparticles	Imaging-PET, MRI	Robert B. Campbell, Srinivas Sridhar

All the data are obtained from Wipo and Google patent.

**Table 3:** Preclinical studies utilizing phytochemical nanocarriers for breast cancer treatment.

Phytochemical Constituent	Anticancer Agent	Nanocarrier	Condition	Remarks	Reference
6-Gingerol	Paclitaxel	PEGylated naniosome	In vivo	Increased the effectiveness of paclitaxel, and lower dose of paclitaxel is needed for the anti-neoplastic activity.	[146]
Quercetin	Doxorubicin	Lecithin	In vivo	Prevents doxorubicin resistance in tumor cells and increases drug absorption and toxicities in malignant cells.	[110]
Quercetin	Doxorubicin	Au nanocages	In vitro	Gives synergistic effect by retaining the drug for longer period of time in malignant cells.	[147]

**Table 4:** Clinical studies utilizing phytochemical nanocarriers for breast cancer treatment.

Phytochemical Constituent-Based Drug	Nanocarrier	Phase of Clinical Trial	Condition	Remarks	References
Vinorelbine tartrate	Liposomal vinorelbine tartrate	Phase 1	Breast cancer	Inhibits microtubule polymerization and promotes cell apoptosis.	[148]
Paclitaxel	Albumin-stabilized paclitaxel	Phase 3	Metastatic breast cancer	Less exposure of toxic cremophor of the drug to non-cancerous cells thus enables higher dosing and improves paclitaxel penetration inside the cancer cells	[149]
	Paclitaxel-loaded polymeric nanoparticles	Phase 4	Breast cancer	Increased blood stability and tumor-specific action by releasing drug inside tumor cells via a PH-sensitive action	[150]
Docetaxel	Nanosomal docetaxel lipid suspension	Phase 3	Breast cancer	Better stability, lower cytotoxicity to normal cells and easily pass-through leaky vasculature of tumor blood vessels	[151]

## References

[1] Bray F., Ferlay J., Soerjomataram I., Siegel L., Torre A., Ahmedin D. (2021). GLOBOCAN estimates of incidence and mortality worldwide for 36 cancers in 185 countries. Glob. Cancer Stat..

[2] Greco S.J. (2019). Breast cancer risk in a rapidly aging population: Advances and approaches to study the aging tissue microenvironment. Breast Cancer Targets Ther..

[3] Arnold M., Morgan E., Rumgay H., Mafra A., Singh D., Laversanne M., Vignat J., Gralow J.R., Cardoso F., Siesling S. (2022). Current and future burden of breast cancer: Global statistics for 2020 and 2040. Breast.

[4] Shnoudeh A.J., Qadumii L., Zihlif M., Al-Ameer H.J., Salou R.A., Jaber A.Y., Hamad I. (2022). Green Synthesis of Gold, Iron and Selenium Nanoparticles Using Phytoconstituents: Preliminary Evaluation of Antioxidant and Biocompatibility Potential. Molecules.

[5] Turashvili G., Brogi E. (2017). Tumor heterogeneity in breast cancer. Front. Med..

[6] Hawkins R., Killen E., Tesdale A., Sangster K., Thomson M., Steele R., Blackie R. (1988). Oestrogen receptors, lactate dehydrogenase and cellularity in human breast cancer. Clin. Chim. Acta.

[7] WHO Classification of Tumours of the Breast. https://espace.library.uq.edu.au/view/UQ:8984059.

[8] Dean-Colomb W., Esteva F.J. (2008). Her2-positive breast cancer: Herceptin and beyond. Eur. J. Cancer.

[9] Bardou V.-J., Arpino G., Elledge R.M., Osborne C.K., Clark G.M. (2003). Progesterone receptor status significantly improves outcome prediction over estrogen receptor status alone for adjuvant endocrine therapy in two large breast cancer databases. J. Clin. Oncol..

[10] Harvey J.M., Clark G.M., Osborne C.K., Allred D.C. (1999). Estrogen receptor status by immunohistochemistry is superior to the ligand-binding assay for predicting response to adjuvant endocrine therapy in breast cancer. J. Clin. Oncol..

[11] Sørlie T., Perou C.M., Tibshirani R., Aas T., Geisler S., Johnsen H., Hastie T., Eisen M.B., Van De Rijn M., Jeffrey S.S. (2001). Gene expression patterns of breast carcinomas distinguish tumor subclasses with clinical implications. Proc. Natl. Acad. Sci. USA.

[12] Sørlie T., Borgan E., Myhre S., Vollan H.K., Russnes H., Zhao X., Nilsen G., Lingjærde O.C., Børresen-Dale A.-L., Rødland E. (2010). The importance of gene-centring microarray data. Lancet Oncol..

[13] Bhushan A., Gonsalves A., Menon J.U. (2021). Current state of breast cancer diagnosis, treatment, and theranostics. Pharmaceutics.

[14] Galmarini D., Galmarini C.M., Galmarini F.C. (2012). Cancer chemotherapy: A critical analysis of its 60 years of history. Crit. Rev. Oncol. Hematol..

[15] Lall R.K., Syed D.N., Adhami V.M., Khan M.I., Mukhtar H. (2015). Dietary polyphenols in prevention and treatment of prostate cancer. Int. J. Mol. Sci..

[16] Sawanny R., Pramanik S., Agarwal U. (2021). Role of Phytochemicals in the Treatment of Breast Cancer: Natural Swords Battling Cancer Cells. Curr. Cancer Ther. Rev..

[17] Ranjan A., Ramachandran S., Gupta N., Kaushik I., Wright S., Srivastava S., Das H., Srivastava S., Prasad S., Srivastava S.K. (2019). Role of phytochemicals in cancer prevention. Int. J. Mol. Sci..

[18] DiMarco-Crook C., Xiao H. (2015). Diet-based strategies for cancer chemoprevention: The role of combination regimens using dietary bioactive components. Annu. Rev. Food Sci. Technol..

[19] Ruiz R.B., Hernández P.S. (2016). Cancer chemoprevention by dietary phytochemicals: Epidemiological evidence. Maturitas.

[20] Bhattacharjee H., Balabathula P., Wood G.C. (2010). Targeted nanoparticulate drug-delivery systems for treatment of solid tumors: A review. Ther. Deliv..

[21] Navya P., Kaphle A., Srinivas S., Bhargava S.K., Rotello V.M., Daima H.K. (2019). Current trends and challenges in cancer management and therapy using designer nanomaterials. Nano Converg..

[22] Duan X., Li Y. (2013). Physicochemical characteristics of nanoparticles affect circulation, biodistribution, cellular internalization, and trafficking. Small.

[23] Pérez-Herrero E., Fernández-Medarde A. (2015). Advanced targeted therapies in cancer: Drug nanocarriers, the future of chemotherapy. Eur. J. Pharm. Biopharm..

[24] Chemotherapy for Breast Cancer. https://www.mayoclinic.org/tests-procedures/chemotherapy-for-breast-cancer/about/pac-20384931.

[25] Grobmyer S.R., Zhou G., Gutwein L.G., Iwakuma N., Sharma P., Hochwald S.N. (2012). Nanoparticle delivery for metastatic breast cancer. Nanomed. Nanotechnol. Biol. Med..

[26] Davies E., Hiscox S. (2011). New therapeutic approaches in breast cancer. Maturitas.

[27] Singh S.K., Singh S., Lillard J.W., Singh R. (2017). Drug delivery approaches for breast cancer. Int. J. Nanomed..

[28] Eroles P., Bosch A., Pérez-Fidalgo J.A., Lluch A. (2012). Molecular biology in breast cancer: Intrinsic subtypes and signaling pathways. Cancer Treat. Rev..

[29] Zhang S., Chu Z., Yin C., Zhang C., Lin G., Li Q. (2013). Controllable drug release and simultaneously carrier decomposition of SiO_2_-drug composite nanoparticles. J. Am. Chem. Soc..

[30] Beloqui A., Alhouayek M., Carradori D., Vanvarenberg K., Muccioli G.G., Cani P.D., Preat V. (2016). A mechanistic study on nanoparticle-mediated glucagon-like peptide-1 (GLP-1) secretion from enteroendocrine L cells. Mol. Pharm..

[31] Yuan H., Ma Q., Ye L., Piao G. (2016). The traditional medicine and modern medicine from natural products. Molecules.

[32] India Regulatory Services > Phytopharmaceutical Drug—CliniExperts. https://cliniexperts.com/india-regulatory-services/phytopharmaceutical-drug/.

[33] Singh B. (2016). Herbal insecticides, Repellents Biomedicines: Effectiveness Commercialization.

[34] Boon H.S., Olatunde F., Zick S.M. (2007). Trends in complementary/alternative medicine use by breast cancer survivors: Comparing survey data from 1998 and 2005. BMC Women’s Health.

[35] O’brien K. (2004). Complementary and alternative medicine: The move into mainstream health care. Clin. Exp. Optom..

[36] Ma H., Carpenter C.L., Sullivan-Halley J., Bernstein L. (2011). The roles of herbal remedies in survival and quality of life among long-term breast cancer survivors-results of a prospective study. BMC Cancer.

[37] Ho J.W., Leung Y., Chan C. (2002). Herbal medicine in the treatment of cancer. Curr. Med. Chem.-Anti-Cancer Agents.

[38] Bahmani M., Shirzad H., Shahinfard N., Sheivandi L., Rafieian-Kopaei M.J. (2017). Cancer phytotherapy: Recent views on the role of antioxidant and angiogenesis activities. J. Evid.-Based Complement. Altern. Med..

[39] Musthaba S.M., Baboota S., Ahmed S., Ahuja A., Ali J. (2009). Status of novel drug delivery technology for phytotherapeutics. Expert Opin. Drug Deliv..

[40] Feng-Lin Y., Tzu-Hui W., Liang-Tzung L., Thau-Ming C., Chun-Ching L. (2008). Preparation and characterization of *Cuscuta chinensis* nanoparticles. Food Chem. Toxicol..

[41] Caiolfa V., Zamai M., Fiorino A., Frigerio E., Pellizzoni C., d’Argy R., Ghiglieri A., Castelli M., Farao M., Pesenti E. (2000). Polymer-bound camptothecin: Initial biodistribution and antitumour activity studies. J. Control. Release.

[42] Min K.H., Park K., Kim Y.-S., Bae S.M., Lee S., Jo H.G., Park R.-W., Kim I.-S., Jeong S.Y., Kim K. (2008). Hydrophobically modified glycol chitosan nanoparticles-encapsulated camptothecin enhance the drug stability and tumor targeting in cancer therapy. J. Control. Release.

[43] Sánchez A., Mejía S.P., Orozco J. (2020). Recent advances in polymeric nanoparticle-encapsulated drugs against intracellular infections. Molecules.

[44] Dong Y., Fu R., Yang J., Ma P., Liang L., Mi Y., Fan D. (2019). Folic acid-modified ginsenoside Rg5-loaded bovine serum albumin nanoparticles for targeted cancer therapy in vitro and in vivo. Int. J. Nanomed..

[45] Kumari M., Sharma N., Manchanda R., Gupta N., Syed A., Bahkali A.H., Nimesh S. (2021). PGMD/curcumin nanoparticles for the treatment of breast cancer. Sci. Rep..

[46] Zeng L., Yan J., Luo L., Ma M., Zhu H. (2017). Preparation and characterization of (−)-Epigallocatechin-3-gallate (EGCG)-loaded nanoparticles and their inhibitory effects on Human breast cancer MCF-7 cells. Sci. Rep..

[47] Solanki R., Patel K., Patel S.J.C., Physicochemical S.A., Aspects E. (2021). Bovine serum albumin nanoparticles for the efficient delivery of berberine: Preparation, characterization and in vitro biological studies. Colloids Surf. A Physicochem. Eng. Asp..

[48] Sulaiman G.M., Jabir M.S., Hameed A.H. (2018). Nanoscale modification of chrysin for improved of therapeutic efficiency and cytotoxicity. Artif. Cells Nanomed. Biotechnol..

[49] Zhou Y., Chen D., Xue G., Yu S., Yuan C., Huang M., Jiang L. (2020). Improved therapeutic efficacy of quercetin-loaded polymeric nanoparticles on triple-negative breast cancer by inhibiting uPA. RSC Adv..

[50] Wu P., Zhang B., Ocansey D.K.W., Xu W., Qian H. (2021). Extracellular vesicles: A bright star of nanomedicine. Biomaterials.

[51] Ortega A., Martinez-Arroyo O., Forner M.J., Cortes R. (2020). Exosomes as drug delivery systems: Endogenous nanovehicles for treatment of systemic lupus erythematosus. Pharmaceutics.

[52] Chen C., Wang J., Sun M., Li J., Wang H.-M.D. (2022). Toward the next-generation phyto-nanomedicines: Cell-derived nanovesicles (CDNs) for natural product delivery. Biomed. Pharmacother..

[53] Fuhrmann G., Serio A., Mazo M., Nair R., Stevens M.M. (2015). Active loading into extracellular vesicles significantly improves the cellular uptake and photodynamic effect of porphyrins. J. Control. Release.

[54] Zhang X., Zhang H., Gu J., Zhang J., Shi H., Qian H., Wang D., Xu W., Pan J., Santos H.A. (2021). Engineered extracellular vesicles for cancer therapy. Adv. Mater..

[55] Wang J., Li W., Lu Z., Zhang L., Hu Y., Li Q., Du W., Feng X., Jia H., Liu B.-F. (2017). The use of RGD-engineered exosomes for enhanced targeting ability and synergistic therapy toward angiogenesis. Nanoscale.

[56] Herrmann I.K., Wood M.J.A., Fuhrmann G. (2021). Extracellular vesicles as a next-generation drug delivery platform. Nat. Nanotechnol..

[57] Wang K., Ye H., Zhang X., Wang X., Yang B., Luo C., Zhao Z., Zhao J., Lu Q., Zhang H. (2020). An exosome-like programmable-bioactivating paclitaxel prodrug nanoplatform for enhanced breast cancer metastasis inhibition. Biomaterials.

[58] Li L., Liang N., Wang D., Yan P., Kawashima Y., Cui F., Sun S. (2018). Amphiphilic polymeric micelles based on deoxycholic acid and folic acid modified chitosan for the delivery of paclitaxel. Int. J. Mol. Sci..

[59] Munagala R., Aqil F., Jeyabalan J., Gupta R.C. (2016). Bovine milk-derived exosomes for drug delivery. Cancer Lett..

[60] Donoso-Quezada J., Guajardo-Flores D., González-Valdez J. (2020). Enhanced exosome-mediated delivery of black bean phytochemicals (*Phaseolus vulgaris* L.) for cancer treatment applications. Biomed. Pharmacother..

[61] Munagala R., Aqil F., Jeyabalan J., Agrawal A.K., Mudd A.M., Kyakulaga A.H., Singh I.P., Vadhanam M.V., Gupta R.C. (2017). Exosomal formulation of anthocyanidins against multiple cancer types. Cancer Lett..

[62] Kanchanapally R., Khan M.A., Deshmukh S.K., Srivastava S.K., Khushman M., Singh S., Singh A.P. (2020). Exosomal formulation escalates cellular uptake of honokiol leading to the enhancement of its antitumor efficacy. ACS Omega.

[63] Antimisiaris S., Marazioti A., Kannavou M., Natsaridis E., Gkartziou F., Kogkos G., Mourtas S. (2021). Overcoming barriers by local drug delivery with liposomes. Adv. Drug Deliv. Rev..

[64] Deshmukh P.K., Mutha R.E., Surana S.J. (2021). Electrostatic deposition assisted preparation, characterization and evaluation of chrysin liposomes for breast cancer treatment. Drug Dev. Ind. Pharm..

[65] Patel G., Thakur N.S., Kushwah V., Patil M.D., Nile S.H., Jain S., Banerjee U.C., Kai G. (2020). Liposomal delivery of mycophenolic acid with quercetin for improved breast cancer therapy in SD rats. Front. Bioeng. Biotechnol..

[66] Farcas C.G., Dehelean C., Pinzaru I.A., Mioc M., Socoliuc V., Moaca E.-A., Avram S., Ghiulai R., Coricovac D., Pavel I. (2020). Thermosensitive betulinic acid-loaded magnetoliposomes: A promising antitumor potential for highly aggressive human breast adenocarcinoma cells under hyperthermic conditions. Int. J. Nanomed..

[67] Zhao Y., Cao Y., Sun J., Liang Z., Wu Q., Cui S., Zhi D., Guo S., Zhen Y., Zhang S. (2020). Anti-breast cancer activity of resveratrol encapsulated in liposomes. J. Mater. Chem. B.

[68] Zawilska P., Machowska M., Wisniewski K., Grynkiewicz G., Hrynyk R., Rzepecki R., Gubernator J. (2021). Novel pegylated liposomal formulation of docetaxel with 3-n-pentadecylphenol derivative for cancer therapy. Eur. J. Pharm. Sci..

[69] García-Pinel B., Porras-Alcalá C., Ortega-Rodríguez A., Sarabia F., Prados J., Melguizo C., López-Romero J.M. (2019). Lipid-based nanoparticles: Application and recent advances in cancer treatment. Nanomaterials.

[70] Wang L., Li H., Wang S., Liu R., Wu Z., Wang C., Wang Y., Chen M. (2014). Enhancing the antitumor activity of berberine hydrochloride by solid lipid nanoparticle encapsulation. AAPS PharmSciTech.

[71] Wang W., Chen T., Xu H., Ren B., Cheng X., Qi R., Liu H., Wang Y., Yan L., Chen S. (2018). Curcumin-loaded solid lipid nanoparticles enhanced anticancer efficiency in breast cancer. Molecules.

[72] Wang W., Zhang L., Chen T., Guo W., Bao X., Wang D., Ren B., Wang H., Li Y., Wang Y. (2017). Anticancer effects of resveratrol-loaded solid lipid nanoparticles on human breast cancer cells. Molecules.

[73] Niazvand F., Orazizadeh M., Khorsandi L., Abbaspour M., Mansouri E., Khodadadi A. (2019). Effects of quercetin-loaded nanoparticles on MCF-7 human breast cancer cells. Medicina.

[74] Radhakrishnan R., Kulhari H., Pooja D., Gudem S., Bhargava S., Shukla R., Sistla R. (2016). Encapsulation of biophenolic phytochemical EGCG within lipid nanoparticles enhances its stability and cytotoxicity against cancer. Chem. Phys. Lipids.

[75] Opatha S.A.T., Titapiwatanakun V., Chutoprapat R. (2020). Transfersomes: A promising nanoencapsulation technique for transdermal drug delivery. Pharmaceutics.

[76] Rai S., Pandey V., Rai G. (2017). Transfersomes as versatile and flexible nano-vesicular carriers in skin cancer therapy: The state of the art. Nano Rev. Exp..

[77] Sivannarayana P., Rani A.P., Saikishore V., VenuBabu C., SriRekha V. (2012). Transfersomes: Ultra deformable vesicular carrier systems in transdermal drug delivery system. Res. J. Pharm. Dos. Technol..

[78] Cevc G., Schätzlein A., Richardsen H. (2002). Ultradeformable lipid vesicles can penetrate the skin and other semi-permeable barriers unfragmented. Evidence from double label CLSM experiments and direct size measurements. Biochim. Biophys. Acta-Biomembr..

[79] Kim B., Cho H.-E., Moon S.H., Ahn H.-J., Bae S., Cho H.-D., An S. (2020). Transdermal delivery systems in cosmetics. Biomed. Dermatol..

[80] Kumar G.P., Rajeshwarrao P. (2011). Nonionic surfactant vesicular systems for effective drug delivery—An overview. Acta Pharm. Sin. B.

[81] Chauhan P., Tyagi B.K. (2018). Herbal novel drug delivery systems and transfersomes. J. Drug Deliv. Ther..

[82] Modi C., Bharadia P. (2012). Transfersomes: New dominants for transdermal drug delivery. Am. J. PharmTech Res. AJPTR.

[83] Li J., Wang X., Zhang T., Wang C., Huang Z., Luo X., Deng Y. (2015). A review on phospholipids and their main applications in drug delivery systems. Asian J. Pharm. Sci..

[84] Moawad F.A., Ali A.A., Salem H.F. (2017). Nanotransfersomes-loaded thermosensitive in situ gel as a rectal delivery system of tizanidine HCl: Preparation, in vitro and in vivo performance. Drug Deliv..

[85] Bnyan R., Khan I., Ehtezazi T., Saleem I., Gordon S., O’Neill F., Roberts M. (2018). Surfactant effects on lipid-based vesicles properties. J. Pharm. Sci..

[86] Gadag S., Narayan R., Sabhahit J.N., Hari G., Nayak Y., Pai K.S.R., Garg S., Nayak U.Y. (2022). Transpapillary iontophoretic delivery of resveratrol loaded transfersomes for localized delivery to breast cancer. Biomater. Adv..

[87] Touitou E., Dayan N., Bergelson L., Godin B., Eliaz M.J. (2000). Ethosomes—Novel vesicular carriers for enhanced delivery: Characterization and skin penetration properties. J. Control. Release.

[88] Godin B., Touitou E. (2003). Ethosomes: New prospects in transdermal delivery. Crit. Rev. Ther. Drug Carr. Syst..

[89] Ainbinder D., Touitou E. (2005). Testosterone ethosomes for enhanced transdermal delivery. Drug Deliv..

[90] Lopez-Pinto J., Gonzalez-Rodriguez M., Rabasco A. (2005). Effect of cholesterol and ethanol on dermal delivery from DPPC liposomes. Int. J. Pharm..

[91] Touitou E. (1996). Compositions for Applying Active Substances to or through the Skin. U.S. Patent.

[92] Touitou E. (1998). Composition for Applying Active Substances to or through the Skin. U.S. Patent.

[93] Nasri S., Ebrahimi-Hosseinzadeh B., Rahaie M., Hatamian-Zarmi A., Sahraeian R. (2020). Thymoquinone-loaded ethosome with breast cancer potential: Optimization, in vitro and biological assessment. J. Nanostruct. Chem..

[94] Barani M., Mirzaei M., Torkzadeh-Mahani M., Adeli-Sardou M. (2019). Evaluation of carum-loaded niosomes on breast cancer cells: Physicochemical properties, in vitro cytotoxicity, flow cytometric, DNA fragmentation and cell migration assay. Sci. Rep..

[95] Barani M., Mirzaei M., Torkzadeh-Mahani M., Nematollahi M.H. (2018). Lawsone-loaded Niosome and its antitumor activity in MCF-7 breast Cancer cell line: A Nano-herbal treatment for Cancer. DARU J. Pharm. Sci..

[96] Honarvari B., Karimifard S., Akhtari N., Mehrarya M., Moghaddam Z.S., Ansari M.J., Jalil A.T., Matencio A., Trotta F., Yeganeh F.E. (2022). Folate-targeted curcumin-loaded niosomes for site-specific delivery in breast cancer treatment: In silico and In vitro study. Molecules.

[97] Ahmed S., Ahmad M., Swami B.L., Ikram S. (2016). A review on plants extract mediated synthesis of silver nanoparticles for antimicrobial applications: A green expertise. J. Adv. Res..

[98] Kuppusamy P., Yusoff M.M., Maniam G.P., Govindan N. (2016). Biosynthesis of metallic nanoparticles using plant derivatives and their new avenues in pharmacological applications–An updated report. Saudi Pharm. J..

[99] Rai M., Yadav A. (2013). Plants as potential synthesiser of precious metal nanoparticles: Progress and prospects. IET Nanobiotechnol..

[100] Gardea-Torresdey J.L., Parsons J., Gomez E., Peralta-Videa J., Troiani H., Santiago P., Yacaman M.J. (2002). Formation and growth of Au nanoparticles inside live alfalfa plants. Nano Lett..

[101] Haverkamp R.G., Marshall A.T., van Agterveld D. (2007). Pick your carats: Nanoparticles of gold–silver–copper alloy produced in vivo. J. Nanopart. Res..

[102] Harris A.T., Bali R. (2008). On the formation and extent of uptake of silver nanoparticles by live plants. J. Nanopart. Res..

[103] Ankamwar B. (2010). Biosynthesis of gold nanoparticles (green-gold) using leaf extract of *Terminalia catappa*. E-J. Chem..

[104] Zhang M., Viennois E., Xu C., Merlin D. (2016). Plant derived edible nanoparticles as a new therapeutic approach against diseases. Tissue Barriers.

[105] Zhuang X., Deng Z.-B., Mu J., Zhang L., Yan J., Miller D., Feng W., McClain C.J., Zhang H.-G. (2015). Ginger-derived nanoparticles protect against alcohol-induced liver damage. J. Extracell. Vesicles.

[106] Li Z., Wang H., Yin H., Bennett C., Zhang H.-G., Guo P. (2018). Arrowtail RNA for ligand display on ginger exosome-like nanovesicles to systemic deliver siRNA for cancer suppression. Sci. Rep..

[107] Akuma P., Okagu O.D., Udenigwe C.C. (2019). Naturally occurring exosome vesicles as potential delivery vehicle for bioactive compounds. Front. Sustain. Food Syst..

[108] Wang Q., Ren Y., Mu J., Egilmez N.K., Zhuang X., Deng Z., Zhang L., Yan J., Miller D., Zhang H.-G. (2015). Grapefruit-Derived Nanovectors Use an Activated Leukocyte Trafficking Pathway to Deliver Therapeutic Agents to Inflammatory Tumor SitesHijacked Leukocyte Pathway for Targeted Delivery. Cancer Res..

[109] Yi S., Wang Y., Huang Y., Xia L., Sun L., Lenaghan S.C., Zhang M. (2014). Tea nanoparticles for immunostimulation and chemo-drug delivery in cancer treatment. J. Biomed. Nanotechnol..

[110] Minaei A., Sabzichi M., Ramezani F., Hamishehkar H., Samadi N. (2016). Co-delivery with nano-quercetin enhances doxorubicin-mediated cytotoxicity against MCF-7 cells. Mol. Biol. Rep..

[111] Halder A., Jethwa M., Mukherjee P., Ghosh S., Das S., Helal Uddin A., Mukherjee A., Chatterji U., Roy P. (2020). Lactoferrin-tethered betulinic acid nanoparticles promote rapid delivery and cell death in triple negative breast and laryngeal cancer cells. Artif. Cells Nanomed. Biotechnol..

[112] Tabassam Q., Mehmood T., Raza A.R., Ullah A., Saeed F., Anjum F.M. (2020). Synthesis, characterization and anti-cancer therapeutic potential of withanolide-A with 20nm sAuNPs conjugates against SKBR3 breast cancer cell line. Int. J. Nanomed..

[113] Zhang N., Yu J., Liu P., Chang J., Ali D., Tian X. (2020). Gold nanoparticles synthesized from *Curcuma wenyujin* inhibits HER-2/neu transcription in breast cancer cells (MDA-MB-231/HER2). Arab. J. Chem..

[114] Mu Q., Wang H., Zhang M. (2017). Nanoparticles for imaging and treatment of metastatic breast cancer. Expert Opin. Drug Deliv..

[115] Al-Mahmood S., Sapiezynski J., Garbuzenko O.B., Minko T. (2018). Metastatic and triple-negative breast cancer: Challenges and treatment options. Drug Deliv. Transl. Res..

[116] Bianchini G., Balko J.M., Mayer I.A., Sanders M.E., Gianni L. (2016). Triple-negative breast cancer: Challenges and opportunities of a heterogeneous disease. Nat. Rev. Clin. Oncol..

[117] Covarrubias G., He F., Raghunathan S., Turan O., Peiris P.M., Schiemann W.P., Karathanasis E. (2019). Effective treatment of cancer metastasis using a dual-ligand nanoparticle. PLoS ONE.

[118] Xu P., Yin Q., Shen J., Chen L., Yu H., Zhang Z., Li Y. (2013). Synergistic inhibition of breast cancer metastasis by silibinin-loaded lipid nanoparticles containing TPGS. Int. J. Pharm..

[119] Christiaens V., Lijnen H.R. (2010). Angiogenesis and development of adipose tissue. Mol. Cell. Endocrinol..

[120] Yoshida S., Ono M., Shono T., Izumi H., Ishibashi T., Suzuki H., Kuwano M. (1997). Involvement of interleukin-8, vascular endothelial growth factor, and basic fibroblast growth factor in tumor necrosis factor alpha-dependent angiogenesis. Mol. Cell. Biol..

[121] Rocha T.G.R., Lopes S.C.D.A., Cassali G.D., Ferreira Ê., Veloso E.S., Leite E.A., Braga F.C., Ferreira L.A.M., Balvay D., Garofalakis A. (2016). Evaluation of antitumor activity of long-circulating and pH-sensitive liposomes containing ursolic acid in animal models of breast tumor and gliosarcoma. Integr. Cancer Ther..

[122] Abraham B.K., Fritz P., McClellan M., Hauptvogel P., Athelogou M., Brauch H. (2005). Prevalence of CD44+/CD24−/low cells in breast cancer may not be associated with clinical outcome but may favor distant metastasis. Clin. Cancer Res..

[123] Charafe-Jauffret E., Ginestier C., Iovino F., Tarpin C., Diebel M., Esterni B., Houvenaeghel G., Extra J.-M., Bertucci F., Jacquemier J. (2010). Aldehyde dehydrogenase 1–Positive cancer stem cells mediate metastasis and poor clinical outcome in inflammatory breast cancer. Clin. Cancer Res..

[124] Singh P., Sahoo S.K. (2022). Piperlongumine loaded PLGA nanoparticles inhibit cancer stem-like cells through modulation of STAT3 in mammosphere model of triple negative breast cancer. Int. J. Pharm..

[125] Tang S.-M., Deng X.-T., Zhou J., Li Q.-P., Ge X.-X., Miao L. (2020). Pharmacological basis and new insights of quercetin action in respect to its anti-cancer effects. Biomed. Pharmacother..

[126] Reyes-Farias M., Carrasco-Pozo C. (2019). The anti-cancer effect of quercetin: Molecular implications in cancer metabolism. Int. J. Mol. Sci..

[127] Aggarwal V., Tuli H.S., Tania M., Srivastava S., Ritzer E.E., Pandey A., Aggarwal D., Barwal T.S., Jain A., Kaur G. (2022). Molecular mechanisms of action of epigallocatechin gallate in cancer: Recent trends and advancement. Semin. Cancer Biol..

[128] Subramani R., Gonzalez E., Arumugam A., Nandy S., Gonzalez V., Medel J., Camacho F., Ortega A., Bonkoungou S., Narayan M. (2016). Nimbolide inhibits pancreatic cancer growth and metastasis through ROS-mediated apoptosis and inhibition of epithelial-to-mesenchymal transition. Sci. Rep..

[129] Lin H., Qiu S., Xie L., Liu C., Sun S. (2017). Nimbolide suppresses non-small cell lung cancer cell invasion and migration via manipulation of DUSP4 expression and ERK1/2 signaling. Biomed. Pharmacother..

[130] Sophia J., Kowshik J., Dwivedi A., Bhutia S.K., Manavathi B., Mishra R., Nagini S. (2018). Nimbolide, a neem limonoid inhibits cytoprotective autophagy to activate apoptosis via modulation of the PI3K/Akt/GSK-3β signalling pathway in oral cancer. Cell Death Dis..

[131] Balakrishnan S., Bhat F., Raja Singh P., Mukherjee S., Elumalai P., Das S., Patra C., Arunakaran J. (2016). Gold nanoparticle–conjugated quercetin inhibits epithelial–mesenchymal transition, angiogenesis and invasiveness via EGFR/VEGFR-2-mediated pathway in breast cancer. Cell Prolif..

[132] Mukherjee S., Chowdhury D., Kotcherlakota R., Patra S., Vinothkumar B., Bhadra M.P., Sreedhar B., Patra C.R. (2014). Potential theranostics application of bio-synthesized silver nanoparticles (4-in-1 system). Theranostics.

[133] Gulia K., James A., Pandey S., Dev K., Kumar D., Sourirajan A. (2022). Bio-Inspired Smart Nanoparticles in Enhanced Cancer Theranostics and Targeted Drug Delivery. J. Funct. Biomater..

[134] Dwivedi A.D., Gopal K. (2011). Plant-mediated biosynthesis of silver and gold nanoparticles. J. Biomed. Nanotechnol..

[135] Cavalcanti I.D.L., Ximenes R.M., Pessoa O.D.L., Magalhães N.S.S., de Britto Lira-Nogueira M.C. (2021). Fucoidan-coated PIBCA nanoparticles containing oncocalyxone A: Activity against metastatic breast cancer cells. J. Drug Deliv. Sci. Technol..

[136] Awad A.B., Williams H., Fink C.S. (2001). Phytosterols reduce in vitro metastatic ability of MDA-MB-231 human breast cancer cells. Nutr. Cancer.

[137] Hamad F.B., Mubofu E.B. (2015). Potential biological applications of bio-based anacardic acids and their derivatives. Int. J. Mol. Sci..

[138] Kushwah V., Katiyar S.S., Dora C.P., Agrawal A.K., Lamprou D.A., Gupta R.C., Jain S. (2018). Co-delivery of docetaxel and gemcitabine by anacardic acid modified self-assembled albumin nanoparticles for effective breast cancer management. Acta Biomater..

[139] Iwanowycz S., Wang J., Hodge J., Wang Y., Yu F., Fan D. (2016). Emodin Inhibits Breast Cancer Growth by Blocking the Tumor-Promoting Feedforward Loop between Cancer Cells and MacrophagesEmodin Blocks Cancer Cell–Macrophage Interaction. Mol. Cancer Ther..

[140] Roopan S.M., Surendra T.V., Elango G., Kumar S.H.S. (2014). Biosynthetic trends and future aspects of bimetallic nanoparticles and its medicinal applications. Appl. Microbiol. Biotechnol..

[141] Wu P., Gao Y., Zhang H., Cai C. (2012). Aptamer-guided silver–gold bimetallic nanostructures with highly active surface-enhanced raman scattering for specific detection and near-infrared photothermal therapy of human breast cancer cells. Anal. Chem..

[142] Przystupski D., Niemczura M.J., Górska A., Supplitt S., Kotowski K., Wawryka P., Rozborska P., Woźniak K., Michel O., Kiełbik A. (2019). In search of Panacea—Review of recent studies concerning nature-derived anticancer agents. Nutrients.

[143] Wei Q.-Y., He K.-M., Chen J.-L., Xu Y.-M., Lau A.T. (2019). Phytofabrication of nanoparticles as novel drugs for anticancer applications. Molecules.

[144] Lohiya G., Katti D.S. (2021). A synergistic combination of niclosamide and doxorubicin as an efficacious therapy for all clinical subtypes of breast cancer. Cancers.

[145] Sartaj A., Baboota S., Ali J. (2021). Assessment of Combination Approaches of Phytoconstituents with Chemotherapy for the Treatment of Breast Cancer: A Systematic Review. Curr. Pharm. Des..

[146] Wala K., Szlasa W., Sauer N., Kasperkiewicz-Wasilewska P., Szewczyk A., Saczko J., Rembiałkowska N., Kulbacka J., Baczyńska D. (2022). Anticancer Efficacy of 6-Gingerol with Paclitaxel against Wild Type of Human Breast Adenocarcinoma. Molecules.

[147] Zhang Z., Xu S., Wang Y., Yu Y., Li F., Zhu H., Shen Y., Huang S., Guo S. (2018). Near-infrared triggered co-delivery of doxorubicin and quercetin by using gold nanocages with tetradecanol to maximize anti-tumor effects on MCF-7/ADR cells. J. Colloid Interface Sci..

[148] (2012). A Bioequivalence Study of Vinorelbine Tartrate Injectable Emulsion in Patients with Advanced Cancer. https://clinicaltrials.gov/ct2/show/NCT00432562?term=NCT00432562&draw=2&rank=1.

[149] Gradishar W.J., Tjulandin S., Davidson N., Shaw H., Desai N., Bhar P., Hawkins M., O’Shaughnessy J. (2005). Phase III trial of nanoparticle albumin-bound paclitaxel compared with polyethylated castor oil–based paclitaxel in women with breast cancer. J. Clin. Oncol..

[150] Park I.H., Sohn J.H., Kim S.B., Lee K.S., Chung J.S., Lee S.H., Kim T.Y., Jung K.H., Cho E.K., Kim Y.S. (2017). An open-label, randomized, parallel, phase III trial evaluating the efficacy and safety of polymeric micelle-formulated paclitaxel compared to conventional cremophor EL-based paclitaxel for recurrent or metastatic HER2-negative breast cancer. Cancer Res. Treat. Off. J. Korean Cancer Assoc..

[151] Subramanian S., Prasanna R., Biswas G., Das Majumdar S.K., Joshi N., Bunger D., Khan M.A., Ahmad I. (2020). Nanosomal docetaxel lipid suspension-based chemotherapy in breast cancer: Results from a multicenter retrospective study. Breast Cancer Targets Ther..

[152] Al-Hakkani M.F., Gouda G.A., Hassan S.H. (2021). A review of green methods for phyto-fabrication of hematite (α-Fe_2_O_3_) nanoparticles and their characterization, properties, and applications. Heliyon.

[153] Baghbani-Arani F., Movagharnia R., Sharifian A., Salehi S., Shandiz S.A.S. (2017). Photo-catalytic, anti-bacterial, and anti-cancer properties of phyto-mediated synthesis of silver nanoparticles from Artemisia tournefortiana Rchb extract. J. Photochem. Photobiol. B Biol..

[154] Singh A., Dar M.Y., Joshi B., Sharma B., Shrivastava S., Shukla S. (2018). Phytofabrication of silver nanoparticles: Novel drug to overcome hepatocellular ailments. Toxicol. Rep..

[155] Meenakshisundaram S., Krishnamoorthy V., Jagadeesan Y., Vilwanathan R., Balaiah A. (2020). Annona muricata assisted biogenic synthesis of silver nanoparticles regulates cell cycle arrest in NSCLC cell lines. Bioorg. Chem..

[156] Singh D., Chaudhary D., Kumar V., Verma A. (2021). Amelioration of diethylnitrosamine (DEN) induced renal oxidative stress and inflammation by *Carissa carandas* embedded silver nanoparticles in rodents. Toxicol. Rep..

[157] Mohammed A.E., Al-Megrin W.A. (2021). Biological Potential of Silver Nanoparticles Mediated by *Leucophyllum frutescens* and *Russelia equisetiformis* Extracts. Nanomaterials.

[158] Aabed K., Mohammed A.E. (2021). Phytoproduct, Arabic Gum and *Opophytum forsskalii* Seeds for Bio-Fabrication of Silver Nanoparticles: Antimicrobial and Cytotoxic Capabilities. Nanomaterials.

[159] Mirzaie A., Badmasti F., Dibah H., Hajrasouliha S., Yousefi F., Andalibi R., Kashtali A.B., Rezaei A.H., Bakhtiatri R. (2022). Phyto-Fabrication of Silver Nanoparticles Using *Typha azerbaijanensis* Aerial Part and Root Extracts. Iran. J. Public Health.

[160] Chandraker S.K., Lal M., Khanam F., Dhruve P., Singh R.P., Shukla R. (2022). Therapeutic potential of biogenic and optimized silver nanoparticles using *Rubia cordifolia* L. leaf extract. Sci. Rep..

[161] Muhammad W., Khan M.A., Nazir M., Siddiquah A., Mushtaq S., Hashmi S.S., Abbasi B.H. (2019). *Papaver somniferum* L. mediated novel bioinspired lead oxide (PbO) and iron oxide (Fe_2_O_3_) nanoparticles: In-vitro biological applications, biocompatibility and their potential towards HepG2 cell line. Mater. Sci. Eng. C.

[162] Naz S., Islam M., Tabassum S., Fernandes N.F., de Blanco E.J.C., Zia M. (2019). Green synthesis of hematite (α-Fe_2_O_3_) nanoparticles using *Rhus punjabensis* extract and their biomedical prospect in pathogenic diseases and cancer. J. Mol. Struct..

[163] Rath K., Sen S. (2019). Garlic extract based preparation of size controlled superparamagnetic hematite nanoparticles and their cytotoxic applications. Indian J. Biotechnol..

[164] Sharma D., Ledwani L., Mehrotra T., Kumar N., Pervaiz N., Kumar R. (2020). Biosynthesis of hematite nanoparticles using *Rheum emodi* and their antimicrobial and anticancerous effects in vitro. J. Photochem. Photobiol. B Biol..

[165] Miri A., Khatami M., Sarani M. (2020). Biosynthesis, magnetic and cytotoxic studies of hematite nanoparticles. J. Inorg. Organomet. Polym. Mater..

[166] Abbasi B.A., Iqbal J., Khan Z., Ahmad R., Uddin S., Shahbaz A., Zahra S.A., Shaukat M., Kiran F., Kanwal S. (2021). Phytofabrication of cobalt oxide nanoparticles from *Rhamnus virgata* leaves extract and investigation of different bioactivities. Microsc. Res. Tech..

[167] Hosny M., Eltaweil A.S., Mostafa M., El-Badry Y.A., Hussein E.E., Omer A.M., Fawzy M. (2022). Facile synthesis of gold nanoparticles for anticancer, antioxidant applications, and photocatalytic degradation of toxic organic pollutants. ACS Omega.

[168] Vundela S.R., Kalagatur N.K., Nagaraj A., Kadirvelu K., Chandranayaka S., Kondapalli K., Hashem A., Abd Allah E.F., Poda S. (2021). Multi-biofunctional properties of phytofabricated selenium nanoparticles from Carica papaya fruit extract: Antioxidant, antimicrobial, antimycotoxin, anticancer, and biocompatibility. Front. Microbiol..

[169] Fouda A., Al-Otaibi W.A., Saber T., AlMotwaa S.M., Alshallash K.S., Elhady M., Badr N.F., Abdel-Rahman M.A. (2022). Antimicrobial, antiviral, and in-vitro cytotoxicity and mosquitocidal activities of *Portulaca oleracea*-based green synthesis of selenium nanoparticles. J. Funct. Biomater..

